# Pre-symptomatic transcriptome changes during cold storage of chilling sensitive and resistant peach cultivars to elucidate chilling injury mechanisms

**DOI:** 10.1186/s12864-015-1395-6

**Published:** 2015-03-26

**Authors:** Clara Pons Puig, Anurag Dagar, Cristina Marti Ibanez, Vikram Singh, Carlos H Crisosto, Haya Friedman, Susan Lurie, Antonio Granell

**Affiliations:** Department of Postharvest Science of Fresh Produce, Agricultural Research Organization, Volcani Center, P.O. Box 6, Bet Dagan, 50250 Israel; Instituto de Biología Molecular y Celular de Plantas, CSIC-Universidad Politecnica de Valencia, E-48022, Valencia, Spain; Plant Sciences Department, University of California Davis, 1 Shields Ave, Davis, CA 95616 USA

**Keywords:** Rosaceae, Microarray, Peach, Woolliness, Internal browning, Reddening

## Abstract

**Background:**

Cold storage induces chilling injury (CI) disorders in peach fruit (woolliness/mealiness, flesh browning and reddening/bleeding) manifested when ripened at shelf life. To gain insight into the mechanisms underlying CI, we analyzed the transcriptome of ‘Oded’ (high tolerant) and ‘Hermoza’ (relatively tolerant to woolliness, but sensitive to browning and bleeding) peach cultivars at pre-symptomatic stages. The expression profiles were compared and validated with two previously analyzed pools (high and low sensitive to woolliness) from the Pop-DG population. The four fruit types cover a wide range of sensitivity to CI. The four fruit types were also investigated with the ROSMETER that provides information on the specificity of the transcriptomic response to oxidative stress.

**Results:**

We identified quantitative differences in a subset of core cold responsive genes that correlated with sensitivity or tolerance to CI at harvest and during cold storage, and also subsets of genes correlating specifically with high sensitivity to woolliness and browning. Functional analysis indicated that elevated levels, at harvest and during cold storage, of genes related to antioxidant systems and the biosynthesis of metabolites with antioxidant activity correlates with tolerance. Consistent with these results, ROSMETER analysis revealed oxidative stress in ‘Hermoza’ and the progeny pools, but not in the cold resistant ‘Oded’. By contrast, cold storage induced, in sensitivity to woolliness dependant manner, a gene expression program involving the biosynthesis of secondary cell wall and pectins. Furthermore, our results indicated that while ethylene is related to CI tolerance, differential auxin subcellular accumulation and signaling may play a role in determining chilling sensitivity/tolerance. In addition, sugar partitioning and demand during cold storage may also play a role in the tolerance/sensitive mechanism. The analysis also indicates that vesicle trafficking, membrane dynamics and cytoskeleton organization could have a role in the tolerance/sensitive mechanism. In the case of browning, our results suggest that elevated acetaldehyde related genes together with the core cold responses may increase sensitivity to browning in shelf life.

**Conclusions:**

Our data suggest that in sensitive fruit a cold response program is activated and regulated by auxin distribution and ethylene and these hormones have a role in sensitivity to CI even before fruit are cold stored.

**Electronic supplementary material:**

The online version of this article (doi:10.1186/s12864-015-1395-6) contains supplementary material, which is available to authorized users.

## Background

Cold storage is used to delay ripening and decay development of many commodities including peaches. However, low temperature storage of peaches also leads to development of chilling injury (CI) manifested as flesh browning (FB), reddening/bleeding (FBL) and woolliness/mealiness (WLT), which limits storage life. CI develops faster and more intensely when susceptible fruit are stored at temperatures between 2.2 and 7.6°C (killing temperature zone) than when stored at 0°C [1,2]. These symptoms mainly develop during fruit ripening after cold storage, and the problem is not noticed until the fruit reaches customers [3].

WLT has been tied to improper cell wall disassembly [1,2]. In WLT fruit the most easily extractable cell wall pectins (soluble in water) are reduced in amount and are of higher molecular weight and viscosity than in ripened, juicy fruit [4,5]. The degree of methylesterification of pectin is also altered. Cell wall pectin participates in the wall in cell-to-cell adhesion, which is accomplished largely by calcium cross-linking between partially de-methylesterified homogalacturonan in the middle lamella [6]. It has been suggested that changes to pectin metabolism cause WLT either by cell fluids forming calcium-pectate gel complexes with high molecular weight pectin in the middle lamella [7], or that the decreased intercellular adhesion in WLT fruit reduces cell rupture during biting and chewing, preventing release of cellular contents [8].

The appearance of FB in the fruit flesh is thought to be related to tissue deterioration or senescence, which leads to changes in membrane permeability and the interaction between phenols and polyphenol oxidase (PO), which are generally found in separate compartments in the cell. Kader and Chordas [9] found that the browning potential of peaches depended on the total amount of phenolic compounds present in the fruit and the level of activity of PO.

FBL has not been studied in depth, but appears to have a large genetic component [1,2]. The symptoms are the dispersion of the anthocyanin pigment which is usually confined to an area next to the pit into the surrounding fruit flesh. Although this is classed as a chilling related disorder, it does not lead to off-flavors or changes in the fruit texture. Current breeding programs include the development of a red fleshed peach, since this will increase the nutritive value of the fruit [10].

Although each of these disorders will develop during cold storage of peaches they have different etiology, and may develop at the same or after different times of storage. Since the underlying molecular pathways of these disorders are different, unravelling and identifying the changes in gene expression leading to each symptom of chilling injury is complicated. What complicates matters more is that the symptoms of CI generally only develop during post-storage ripening; therefore fruits that appear healthy at the end of storage may develop one or more of the CI symptoms during post-storage warming.

Modern breeding of peaches started in the USA towards the end of the 19^th^ century and was based on a very limited number of genotypes [11]. Thus, because of this and because of their high degree of natural self-pollination, peach cultivars are known to have low genetic variability [12]. Although the genetic background of peaches is very limited, there are differences between cultivars in their resistance to prolonged cold storage and chilling injury. In an attempt to study the genetic basis for chilling injury, the commercial cultivar ‘Dr. Davis’ was crossed with ‘Georgia Belle’. ‘Dr. Davis’ produces yellow, clingstone, non-melting flesh fruit mainly for the canning industry, while ‘Georgia Belle’ produces white, freestone, melting flesh fruit that are eaten fresh [13-15]. The progeny segregated in their sensitivity to cold storage and the sensitive peaches developed 80% woolliness and the tolerant peaches had no woolliness during ripening after 1 week’s storage at 5°C [15,16].

The present study examines transcriptomic changes while the fruit are still in cold using the Chillpeach microarray (the limitations of this approach are discussed in [17]). In our study we examine harvested non-stored fruit and two time points of unripe stored fruit of two white- melting-flesh cultivars, ‘Oded’ (Od, a cling-stone, early season peach) and ‘Hermoza’ (Hz; free-stone, mid-season peach), with different sensitivity towards CI, in which symptoms of chilling injury (WLT, FB and FBL) are not apparent. Candidate genes associated to tolerance/sensitivity in these fruits were identified and expression of some genes was further validated by quantitative real-time PCR (qRT-PCR). We also validate the results and extend the Pons et al. [17] study by integrating Od and Hz data with expression data of pools of siblings from the Pop-DG population with contrasting sensitivity to WLT using the same sampling times (harvest, 1 and 2 weeks at 5°C). This comparison has enabled us to find (i) genes that respond to cold similarly in all four peach fruit (core cold responses) (ii) but differ in their time/levels of expression and therefore may be directly related to the sensitivity/tolerance to cold and (iii) genes specific for different chilling injury symptoms and/or to the tolerance specific for each fruit cultivar. Furthermore, by using ROSMETER [18] we characterize *in silico* the Reactive Oxygen Species (ROS) signature (ROS types and their subcellular origins) of peach fruit during cold storage.

## Methods

### Fruit material and post-harvest conditions

The experiments were carried out with an early-season variety peach [*Prunus persica (L.)* Batsch ‘Oded’] (Od) and a mid-season variety peach [*Prunus persica (L.)* Batsch ‘Hermoza’] (Hz) in 2009. Fruit of both cultivars were harvested, from a commercial orchard in Israel, at commercial maturity stage (H), according to Kader & Mitchel [19]. Fruit and physiological parameters at harvest are recorded in Table 1. Some fruit were allowed to ripen at shelf life during 3 days at 20°C (SL samples), while the rest were stored immediately at 5°C. The fruit were removed from cold storage every week for up to three weeks (CS samples). Pooled mesocarp tissue from 5 fruit were flash frozen with liquid nitrogen and stored at −80°C until further analysis. After each storage period, some fruit were ripened at 20°C for 3 days (CS + SL) for chilling injury and quality evaluation.

**Table 1:** **Physiological parameters of** ‘**Oded**’ **and** ‘**Hermoza**’ **at harvest**

**Cultivar**	**Weight (** **g)**	**Ethylene** **(μL kg** ^**−1**^ **h** ^**−1**^ **)**	**SSC (%)**	**TA (%)**	**Firmness** **(Newton)**
**Oded**	141 ± 15.0b	0.69 ± 0.53a	11.9 ± 0.90b	0.43 ± 0.05a	54.0 ± 7.2a
**Hermoza**	200 ± 30.6a	0.78 ± 1.00a	14.3 ± 0.46a	0.33 ± 0.03b	62.8 ± 11.2a

Different letters indicate significant differences at P < 0.05 (t-test).

### Physiological parameters

Physiological parameters were measured and averaged from 15 fruit at harvest, after cold storage and after subsequent shelf life ripening following the protocol described in Zhou et al. [5]. Firmness was measured on two pared sides of each fruit using a penetrometer fitted with an 8-mm diameter plunger. A wedge-shaped slice (approx. 5 g) was removed from each fruit in the replicates and the pooled sample was passed through an electric juicer (Moulinex, type 753, France) for the measurement of soluble solids content (SSC) and titratable acidity (TA). SSC was determined by a digital refractometer (Atago, Tokyo, Japan). The TA was determined by titration of 2 mL juice to pH 8.2 with 0.1 N NaOH and expressed as percentage of malic acid. Ethylene was determined by closing individual fruit in a 650 ml jar for 1 h, sampling the air in the container with a syringe and injecting into a gas chromatograph with a FID detector.

### Chilling injury evaluation

Fruit were evaluated for different CI symptoms such as expressible of juice, hard textured fruit with no juice upon squeezing or woolly texture (WLT), flesh browning or pit cavity browning (FB) and internal reddening or flesh bleeding (FBL) after cold storage (CS) and after shelf life ripening during 3 days at 20°C (CS + SL). Observations were made on 15 fruit at each observation time.

WLT was determined in both CS and CS + SL fruit as the amount of expressible juice as described in Dagar et al. [20]. Expressible juice was indicated as the percentage of free juice in total tissue [21]. WLT was also evaluated visually along with FB and FBL in CS + SL fruit. Each fruit was cut into two halves through the suture plane. WLT was scored on a 5-grade scale, according to amount of juice released upon hand squeezing, as follows: 1, very juicy; 1.5, moderate juicy; 2, less juicy; 2.5, small amount of juice; and 3.0, almost no juice. FB and FBL were also scored according to a 5-grade scale, based on area covered as follows: 1, no browning or reddening; 1.5, affected area < 5%; 2, affected area ≥ 5% and < 25%; 2.5, affected area ≥ 25% and < 50%; and 3.0, affected area ≥ 50%. Results for WLT, FB and FBL were expressed as an index calculated as the percentage of the average of fruit with each CI level in the treatment.

### RNA extraction, microarray and data analyses

For the microarray experiments, the mesocarp RNA for each genotype at harvest (H), after cold storage for 1 week (CS1) and 2 weeks (CS2) at 5°C were all converted into labeled cDNA and hybridized to the Chillpeach microarray [16]. All samples were compared using a dye-swap design against the common superpool reference used in Pons et al. [17]. Three replicates from each sample pool were hybridized in each case, one of them dye-swapped.

RNA purification, sample preparation and hybridization to Chillpeach microarray were performed as described in Ogundiwin et al. [16]. To generate raw data to be used for expression analysis Lowess M Log Ratio was used as expression value and patterns with more than 80% of missing values were filtered. In total, 3277 probes met the threshold for hybridization quality. Differentially expressed genes were identified from the raw dataset using Significance Analysis of Microarray software (SAM, [22]) as described in Pons et al. [17]. Statistical significance for global analysis was assessed with a false discovery rate (FDR) of 1%, q-value ≤0.01. A total of 3002 genes showed significance. Significant data were normalized to harvest expression values after removing 38 genes without data in Hz at harvest. A total of 2964 unigenes were used for further analyses. Statistical significances for direct comparisons between cold storage times were assessed with a FDR of 5%, q-value ≤0.05.

Principal component analysis and 2D-hierarchical cluster were performed on significant data using Acuity™ (Axon instruments) as described in Pons et al. [17]. Functional enrichment is performed as described in Pons et al. [17] and results were visualized using, Matrix2png [23].

### Comparison of the cold response of ‘Oded’ (Od), ‘Hermoza’ (Hz) and pools of siblings from the Pop-DG population

In order to examine transcript abundance changes across different peach fruit differing in their sensitivity to chilling injury, and to compare these with the transcript abundance profiles generated from this study, transcriptome data from pools of siblings from the Pop-DG population at harvest and after one and two weeks of cold storage at 5°C were retrieved from Pons et al. [17]. For the comparative analysis genes with high quality values in the two experiments (see above) and differentially expressed between Hz and Od and between highly sensitive (S) and less sensitive (LS) pools after one week of cold storage were selected. A dataset of 2207 genes was generated and used for the comparison. Clustering of total transcript accumulation within a specific treatment and fruit type was done using Euclidean distance and the *k*-means unsupervised clustering Acuity™ (Axon instruments). For calculations the number of k clusters was set to 12 and the centroid for each cluster was randomly assigned. Spots with missing values were replaced with the average values across the arrays. Profiles with the same shape pattern were centered and scaled around the mean value across arrays. Transcripts were ordered in the clusters according to their contribution to principal component 1 of the PCA performed with the same dataset.

### Real-time quantitative reverse transcriptase-PCR analysis

The transcript abundance of 10 selected genes (Additional file 1: Table S1) that were differentially expressed between Od and Hz were validated with quantitative reverse transcriptase polymerase chain reaction (qRT-PCR) analyses. Full length cDNA, primer design, optimum primer and cDNA concentrations, qRT-PCR reaction and quantification was performed as described in Dagar et al. and Pons et al. [17,24]. Primer sequences and amplicon lengths are given in Additional file 1: Table S1. Each biological sample was examined in duplicate with two to three technical replicates. The expression levels for the genes were calculated relative to the Initiation Factor eIF-4-Gamma (eIF-G) gene as described by Ogundiwin et al. [16], and the expression level of each analyzed gene transcript during cold storage in the Od and Hz samples was calculated relative to this harvest values.

### ROSMETER analysis

The ROSMETER is a new bioinformatic tool (http://app.agri.gov.il/noa/ROS_desc.php), which can provide information on the specificity of ROS-related response for any data set [18]. The ROSMETER was fabricated by using data from Arabidopsis plants exposed to stresses occurring in different cellular compartments. A set of genes having Arabidopsis orthologs in Chillpeach [16] and differentially expressed at harvest and during cold storage in all four fruits studies was used for ROSMETER analysis. The obtained data set was arranged according to the instructions on the website and submitted for analysis. The output file represents correlation values between known oxidative stresses and the transcriptome of the two cultivars and the two pools of siblings at harvest and following cold storage of 1 and 2 weeks. Correlation values above 0.12 represent non-random correlations [18].

## Results

### Ripeness and chilling injury parameters of ‘Oded’ and ‘Hermoza’ peaches

The fruits of Od and Hz were slightly different at harvest. Ripening parameters and results of t-tests are summarized in Table 1. At harvest, Od peach fruit were 30% smaller (by weight) than Hz peaches. Furthermore, Hz fruit were less acidic (0.33% compared to 0.43%), and had higher soluble solids (14% compared to 12%). However, there were no significant differences in ethylene production or in firmness between fruit of the two cultivars. The ethylene levels in Od and Hz fruit were 0.69 μL kg^−1^ h^−1^ and 0.78 μL kg^−1^ h^−1^, respectively (Table 1). According to Kader & Mitchell [19] both cultivars were harvested at similar commercial mature stage (M). However it is obvious that physiological differences exist between both cultivars at the mature commercial stage, related to their growing conditions, length of development and genetic background.

**Figure 1:**
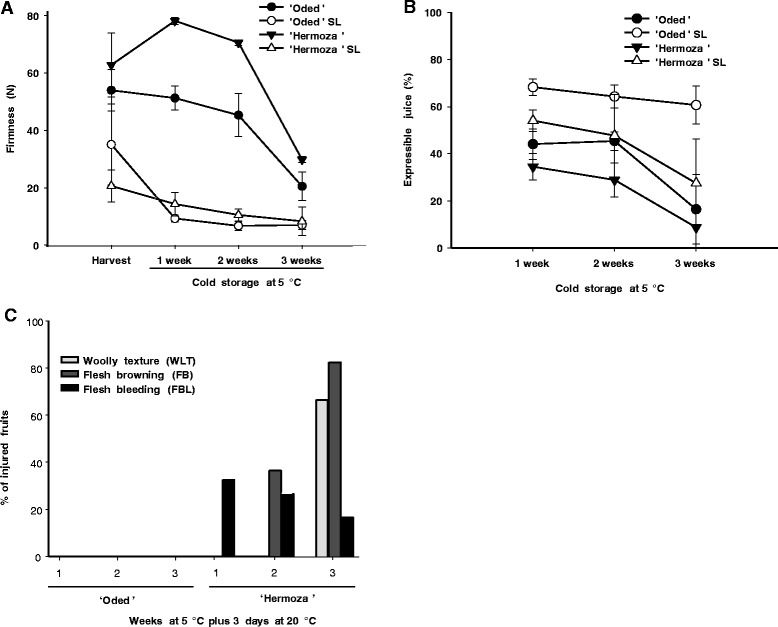
**Comparison of chilling injury symptoms of** **‘Oded’** **and** **‘Hermoza’.** **A)** Firmness of ‘Oded’ and ‘Hermoza’ peaches at harvest and after cold storage at 5°C (black colored symbols), and during ripening at 20°C (shelf life, open circles). Standard deviation is indicated. **B)** Expressible juice of ‘Oded’ and ‘Hermoza’ peaches at harvest and after cold storage at 5°C (black colored symbols), and during ripening at 20°C (shelf life, open symbols). **C)** Woolly texture (WLT), flesh browning (FB) and flesh bleeding (FBL) indices of ‘Hermoza’ peaches during shelf life after cold storage at 5°C.

Although Hz peaches were firmer than Od peaches during storage (Figure 1A), these cultivars exhibited similar trends in firmness during CS at 5°C. Fruit of both cultivars retained their firmness for the first two weeks of storage, and upon the third week in the cold began to soften (Figure 1A). The firmness levels of both cultivars during shelf life (SL) ripening following cold storage (CS), although much lower than during CS, was also similar; with Od reaching 7 to 9 N, and Hz between 8 to 14 N. These values were lower than the softening that occurred when the fruit were held for three days at 20°C without storage (Figure 1A).

Consistent with previous findings that Od fruit were resistant to CI in CS [21], expressible juice did not change during SL ripening after CS (remaining approximately 65%) while Hz decreased to 27% (Figure 1B) and no WLT was observed visually in Od fruit (Figure 1C). Further, there were no symptoms of FB or FBL in Od during ripening after CS for up to three weeks (Figure 1C). In contrast, Hz was sensitive to CI, and developed FB and FBL during SL after 2 weeks of CS and all three CI symptoms after 3 weeks.

### Global transcriptome analysis

The Chillpeach microarray [16] was used to analyze the transcriptomes of peaches from both cultivars at harvest and after 1 and 2 weeks of storage. These stages were selected to investigate pre-symptomatic early events in the chilling response which may be associated to WLT, FBL and FB.

**Figure 2:**
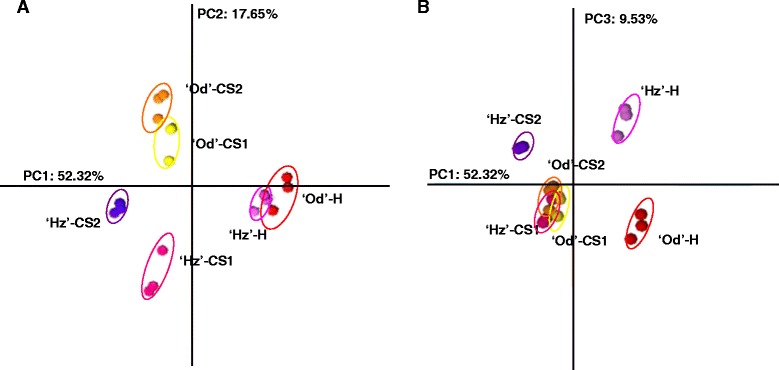
**Principal Component Analysis** **(PCA)** **of harvest and cold stored** **‘Oded’** **and** **‘Hermoza’** **samples according to their lowess normalized expression data.** Three biological replicates per sample were analyzed. **A)** First principal component (PC1) is shown on x-axis while the second principal component (PC2) is shown on y-axis. **B)** PC1 is shown on x-axis while the third principal component (PC3) is shown on y-axis. The percentage of the variance explained by each component is indicated. Od: ‘Oded’; Hz: ‘Hermoza’; H: harvest; CS1: 1 week at 5°C; CS2: 2 weeks at 5°C.

In total, 3277 probes met the threshold for hybridization quality (Additional file 2: Table S2). As a first approach to analyze the complexity of the gene expression dataset, a Principal Component Analysis (PCA) was performed on raw data. The three first components account for 80% of variance (Figure 2A, B). The results of the PCA plot showed consistency across replicated samples and treatments and, therefore, the experiment was considered reliable for further analysis. The 1^st^ component (PC1, 52.32% variance) clearly separated harvest from cold-treated samples (Figure 2A). The 2^nd^ component (PC2, 17.65%) separated cold stored samples of the tolerant cultivar Od from the sensitive Hz. The 3^rd^ component (PC3) which contributed 10% of the difference in gene expression, separated the two cultivars at harvest (Figure 2B), which indicates that most of differences in the transcriptome induced by cold are due to differences in the sensitivity to develop injury rather than to differences at harvest. However, PC3 shows that genes differentially expressed at harvest reach similar expression values after being cold stored 1 and 2 weeks in Od and after 1 week in Hz, but not Hz-CS2 fruit, which were projected separately from the other cold stored samples. This indicates that genes differentially expressed at harvest could be involved in the eventual injury these fruit suffered when shelf ripened after two weeks in the cold (i.e. FB) but not to the phenotypical differences observed by just one week (i.e. FBL).

### Differences in the transcriptome of ‘Oded’ and ‘Hermoza’ fruits at harvest and during cold storage

**Figure 3:**
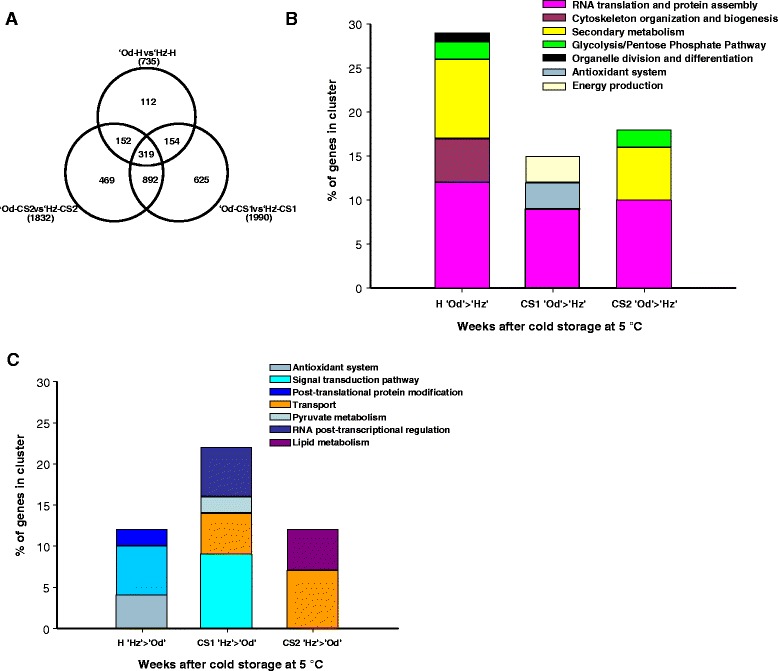
**Differential gene expression between the** **‘Oded’** **and** **‘Hermoza’** **peach fruits at harvest and after 1 and 2 weeks of cold storage. A)** A Venn diagram showing the differentially expressed genes (FDR < 0.05 and q-value < 0.05) between the tolerant Od and the sensitive Hz fruits at each time of cold storage. **B)** The over-represented functional categories (p-value 0.05) corresponding to the differentially expressed genes high expressed in Od comparing to Hz at harvest and at each time of cold storage. **C)** The over-represented functional categories (p-value 0.05) corresponding to the differentially expressed genes high expressed in Hz comparing to Od at harvest and at each time of cold storage. H: Harvest; CS1: cold storage of 1 week at 5°C; CS2: cold storage of 2 weeks at 5°C; Od: ‘Oded’ peach; Hz: ‘Hermoza’ peach.

A direct comparison between Od and Hz peaches at harvest and at the different cold storage periods (CS1 and CS2) was carried out in order to identify genes differentially expressed in between the two cultivars and thus, eventually, to discover genes involved in chilling injury resistance/sensitivity at pre-symptomatic stage. As shown in Figure 3A the number of differentially expressed genes between the two cultivars was higher following cold storage (1 and 2 weeks) than at harvest, thus confirming PCA results.

A total of 735 genes were differentially expressed at harvest, and out of these 344 and 393 genes were up- and down-regulated, respectively, in Od compared with Hz at harvest (Figure 3A; Additional file 3: Table S3). As shown in Figure 3B, the genes with higher expression in Od at harvest were functionally enriched in *RNA translation and protein assembly*, *cytoskeleton organization and biogenesis*, *secondary metabolism*, *glycolysis* and *organelle division and differentiation*. Genes under-represented (i.e., overexpressed in the sensitive cultivar Hz) were enriched in *antioxidant system*, *signal transduction*, *post*-*translational protein modification* and *unknown function* (Figure 3C). Approximately 90% of the genes differentially expressed at harvest have altered expression during cold storage (Figure 3A). This suggests that they have to do with the differential chilling response in both cultivars (as we showed in PCA, Figure 2). However, some of them belonging to functional categories such as *cell wall*, *glycolysis*, *tricarboxylic acid cycle* (*TCA*) and *other carbohydrate metabolism*, and may also account for the physiological differences observed between Od and Hz at harvest (firmness, soluble solid content, acidity; see Table 1; Additional file 3: Table S3). By one week of CS, 1990 genes were differentially expressed (Figure 3A).

Functional enrichment indicated that *RNA translation and protein assembly* was higher in Od than in Hz, both at harvest and during 1 and 2 weeks of cold storage (Figure 3B). Out of 42 genes in this functional class over-represented in Od at harvest, 21 genes were also higher expressed in Od at one week of cold and 12 genes by two weeks. Moreover, 61 genes also showed high expression levels in Od by 1 and 2 weeks of cold storage and twenty genes were common in all three time points (Additional file 3: Table S3). This suggests that enhanced protein synthesis at harvest and during cold is critical for tolerance development. *Secondary metabolism* and *glycolysis* enriched genes were highly expressed in Od both at harvest and after 2 weeks of cold storage (Figure 3B). This overlap indicates that differences at harvest may account for the differences observed at 2 weeks of CS, as suggested the PCA (Figure 2). Genes of the *signal transduction* and *transport* functional categories were enriched in the sensitive cultivar Hz at harvest and also after 1 week of CS (Figure 3C), thus suggesting that they may be related to the sensitivity to cold storage. The functional category *antioxidant systems* was enriched in both cultivars at different time points. Fifteen antioxidant related genes were more highly expressed in Hz at harvest (15 genes) and 30 were over-represented in CS1 of Od peaches (Figure 3B and C). Out of the 15 genes encoding for antioxidant activities, 11 were high expressed in Od peaches at one week. This suggests that high levels of antioxidants at harvest are not directly related to the tolerance to cold storage, rather it appears that high levels of antioxidants during cold storage contribute to the tolerance. In addition, only four genes encoding for antioxidant activities were highly expressed in Hz at harvest and, as is the case of the orthologs of catalase 2 (CAT2) and thioredoxin (TRXH2), also during cold storage (Additional file 3: Table S3), suggesting that they are related to the sensitivity to cold.

### Kinetics of the cold response in ‘Oded’ and ‘Hermoza’

**Figure 4:**
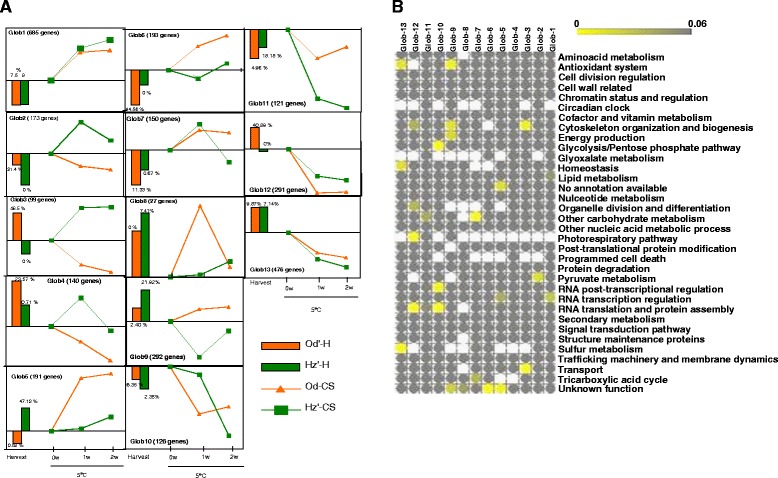
**Kinetics of cold responsive genes in** **‘Oded’** **and** **‘Hermoza’ fruits during cold storage and harvest values. A)** Average gene expression pattern relative to harvest of genes in each of the 13 clusters generated by unsupervised two-dimensional hierarchical clustering (Additional 4: Figure S1). Od and Hz harvest values (bars) represents the average fold change of all genes within a cluster with respect to the reference pool. The percentage of genes high expressed at harvest in each cultivar and cluster is indicated together with expression bars. The number of genes in each cluster is indicated between brackets. **B)** The functional categories overrepresented in each cluster are shown as a heatmap obtained with matrix2png. Enriched functional categories with Fisher test p-values < 0.05 are colored in grades of yellow. Harvest; CS1: cold storage of 1 week at 5°C; CS2: cold storage of 2 weeks at 5°C; Od: ‘Oded’ peach; Hz: ‘Hermoza’ peach.

To investigate chilling-induced alterations in the gene expression profiles of the two cultivars in this study, differentially expressed genes were assessed with a false discovery rate (FDR) of 1%, q-value ≤0.01 based on three replicates. We found 2964 genes differentially regulated at least for one condition (samples H and CS samples) in either of genotypes. To distinguish whether transcripts are differentially affected by cold and analyze kinetics while avoiding the effect of harvest differences, expression data was normalized to harvest values. Differentially expressed transcripts were grouped according to shared cold expression patterns by Hierarchical Cluster Analysis (HCA) (Figure 4A; Additional file 4: Figure S1) and further characterized by functional enrichment (Figure 4B). In order to reflect the expression levels of genes at harvest, the average expression value of all genes in a cluster and the percentage of genes with higher expression levels in each cultivar (from the direct comparison; Figure 3) and each cluster were plotted in the graphic together with the cold expression profile. Only when the percentage of genes more highly expressed in a cultivar exceeded 20% of the genes in a cluster, was their contribution considered significant. The HCA resulted in 13 clusters (Figure 4A). Based on their expression during cold storage, these genes can be classified into several groups as follows.

The largest group (A) comprises cold responsive genes irrespective of sensitivity to cold storage. These included 685 genes (cluster 1, Figure 4A) up regulated by cold storage and enriched in *RNA transcription regulation* (Figure 4B) and 767 genes cold down-regulated (in clusters 12 and 13; Figure 4A) enriched in *cytoskeleton organization*, *organelle division*, *photorespiratory pathway* (cluster 12; Figure 4B) and *antioxidant system*, *homeostasis*, and *sulfur metabolism* (cluster 13; Figure 4B). This indicates that cold storage in both peach cultivars involves the activation of several transcriptional cascades and an extensive down-regulation of housekeeping and metabolic functions. Most of genes in clusters 1 and 13 do not show statistical differences in expression at harvest, while 41% of genes in cluster 12 were highly expressed in Od at harvest (Figure 4A). This suggests that, although the effect of cold on genes in cluster 12 is down-regulation, high levels at harvest can contribute to withstanding cold storage.

The second group (B) includes 538 genes comprised of clusters 2, 3 and 4 that most likely contains genes up-regulated during cold storage in the sensitive cultivar Hz while down-regulated in the tolerant Od (Figure 4A), suggesting a possible relation to chilling sensitivity. Genes in clusters 2 and 3 were enriched in genes related to *pyruvate metabolism*, *cytoskeleton organization* and *transport* (Figure 4B). Genes in cluster 4, which was transiently up-regulated in Hz (Figure 4A) did not show any enrichment. It is noteworthy that 20% of the genes in clusters 2, 3 and 4 clusters were expressed at higher levels in Od cultivar at harvest (Figure 4A), especially genes in cluster 3, where genes with higher expression levels in Od-H account for 46.5% of genes, suggesting that they may be part of a constitutive tolerance mechanism. However, the observation that these genes were cold-induced in the sensitive cultivar Hz indicated that they could be required for setting up the initial response to cold, but do not enable the fruit to stand long term cold periods.

The third group (C) included 797 genes included in clusters 5, 6, 9 and 11 that during cold storage were expressed at higher levels in Od compared to Hz (Figure 4A), and thus may be related with to CI resistance. Clusters 5, 6 and 9 comprised genes up-regulated in Od during CS, but unaffected or even decreased in Hz (Figure 4A). Genes in clusters 5 and 6 were enriched in genes without annotation or with *unknown function*; however class 9 was enriched with genes related to *antioxidant system*, *cytoskeleton organization*, *energy production* and genes of *unknown function* (Figure 4B). More than 20% of genes in these three clusters were expressed at higher levels in the sensitive cultivar before cold stress, but during cold storage most of them reach expression values higher in Od than in Hz (Figure 4A). This suggests that high levels of these genes may contribute to the tolerance to cold storage and that the ability to up-regulate these genes during cold was related to low levels at harvest. The genes of cluster 11, enriched in *other carbohydrate metabolism* (Figure 4B), were down-regulated during cold storage in both cultivars; however the expression levels in Od were always higher than in Hz (Figure 4A). No significant differences were observed at harvest. Interestingly, this cluster (Additional file 3: Table S3) contained the orthologs of CBF1 (C-repeat/DRE Binding Factor 1) and CAMTA2 (Calmodulin Binding Transcription Activator 2), two transcription factors playing important roles during cold acclimation [25,26], thus confirming the possible role of the genes in group C in chilling injury tolerance.

A fourth group (D) was formed by clusters 7, 8 and 10. The genes in these clusters did not show in general differences at harvest, but had the particularity of being transiently up-regulated or maintained at harvest expression level in one of the cultivars (Figure 4A). The genes in cluster 7, enriched in *other carbohydrate metabolism* and *TCA* genes (Figure 4B), were up-regulated to similar rates in both cultivars, but repressed in the sensitive cultivar after two weeks, when browning started to develop when fruit were shelf ripened. This suggests that down-regulation of these genes might be related to the development of injury at a pre-symptomatic stage. The genes in cluster 8, enriched in genes with unknown function, did not respond to cold in Hz but transiently up-regulated in Od, suggesting a possible regulatory role of these genes. Genes in cluster 10 (Figure 4A) which was enriched in *glycolysis*, *RNA posttranslational regulation*, and *RNA translation and protein assembly* (Figure 3B), did not respond to cold in Hz during the first week while being down-regulated in Od from this time (Figure 4A). This suggests that the response to cold of these genes was delayed in the sensitive cultivar Hz which may be counterproductive to withstanding the cold storage.

### Validation of Hz and Od microarray results

**Figure 5:**
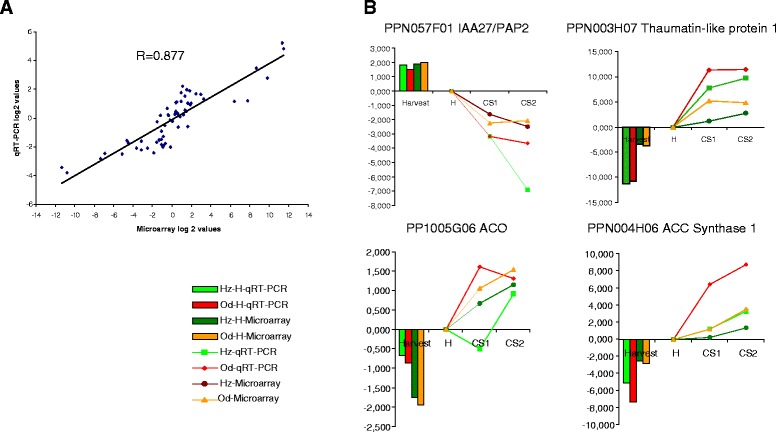
**Quantitative RT**-**PCR validations of microarray data in Oded and Hermoza fruits. A)** The comparison of the microarray and qRT-PCR assay data, based on log2 data in Od and Hz. A total 10 differentially expressed genes were chosen, representing 60 comparisons where one gene covers 3 different time points (H, CS1 and CS2). Line shown represents the orthogonal fit to the data and correlation (R) is shown. **B)** Example of gene expression profiles across H, CS1 and CS2 samples in Od and Hz determined by qRT-PCR and microarray on four peach genes previously associated to CI tolerance (IAA27, Thaumatin-1-like, ACS and ACO). In the graphs there are represented Od and Hz values at harvest (bars) and the average gene expression pattern relative to harvest values in both platforms, microarray and qRT-PCR.

In order to validate the microarray results, we performed qRT-PCR on ten peach genes selected from the list of genes differentially expressed between Od and Hz fruits using gene specific primers (Additional file 1: Table S1). The tested genes were chosen from different processes including *cell wall*, *RNA transcription regulation*, *secondary metabolism*, *signal transduction pathway* and *trafficking machinery and membrane dynamics* (Additional file 5: Table S4). A total of 60 comparisons were made, as the expression of each gene was monitored at three time points (H, CS1 and CS2) in Od and Hz, using the same samples used for microarray analyses. The overall correlation observed between microarray and qRT-PCR analysis was R = 0.88 (Figure 5A). In addition, we also evaluated the agreement between each gene’s expression profiles determined by qRT-PCR and microarrays using Pearson correlation coefficient (Additional file 5: Table S4). The qRT-PCR data correlate well (range R =0.8-1, six genes) or are consistent (range R = 0.5-0.8, four genes) with the patterns of expression revealed by microarray analysis, and four examples (Figure 5B) include those for Thaumatin-like protein 1 (PPN003H07), aminocyclopropane-1-carboxylic acid (ACC) synthase (ACS1; PPN004H06), ACC oxidase (ACO; PP1005G06) and the ortholog of the transcription factor indoleacetic acid-induced protein 27 (IAA27/PAP2; PPN057F01), reported as being associated to woolliness tolerance at a pre-symptomatic stage [17,20,2728]. These results confirm the general validity and robustness of the microarray data we present here.

Another source of validation comes from the microarray-based genome-wide analysis of pools from Pop-DG population with contrasting WLT sensitivity in response to cold storage [17]. The similarity between of Pons et al. [17] data and those presented here for Od and Hz (same developmental stage, treatments as well same expression platform and reference pool for hybridization), allow direct comparison of expression profiles and values between studies.

The pools of the Pop-DG population are less tolerant to WLT than Hz. The most sensitive pool (high sensitive, S) was already mealy/woolly after one week of cold storage at 5°C plus shelf life ripening, while the relative tolerant (low sensitive, LS) was damaged after two weeks of cold storage [17]. However, while Hz was more resistant to WLT (fruit showed WLT symptoms after 3 weeks in cold) the siblings from Pop-DG population were tolerant to FBL and FB [17], which developed in Hz during ripening after two weeks of storage. However if tolerance/sensitive mechanisms are conserved, we expect that genes high expressed in the tolerant Od by compared to Hz, were high expressed in the LS pool compared to the S pool.

We have compiled a dataset of 2207 genes (Additional file 6: Table S5) integrating expression values for cold responsive genes, differentially expressed at one week of cold storage, when the largest number of differentially expressed was found among all fruit. Then we determined the percentage of differentially expressed genes identified in each study (Od vs Hz and LS vs S pools) that shared the expression patterns. The comparison between both experiments resulted in more than 55% of the genes showing consistent patterns of expression (Additional file 6: Table S5). These ‘consistent genes’ corresponded to genes highly expressed in the LS pool that also showed higher expression levels in Od than in Hz, while genes with higher expression in S pool than in the LS pool showed higher expression levels in Hz. The rest of cold responsive genes were only differentially expressed in one of the experiments (20-30%) or showed an opposite pattern (<10%). These observed differences may indicate differences in the response to cold due to cultivar. Nonetheless, considering that 55% of genes had similar transcript profiles across samples and the low proportion of genes behaving in opposite direction, this comparative transcriptomic approach provides a valuable indication of a set of candidate genes that can be related to tolerance/sensitivity to CI in peach.

### Comparison of the transcriptomes of ‘Oded’ and ‘Hermoza’ with Pop-DG siblings with contrasting sensitivity to WLT

**Figure 6:**
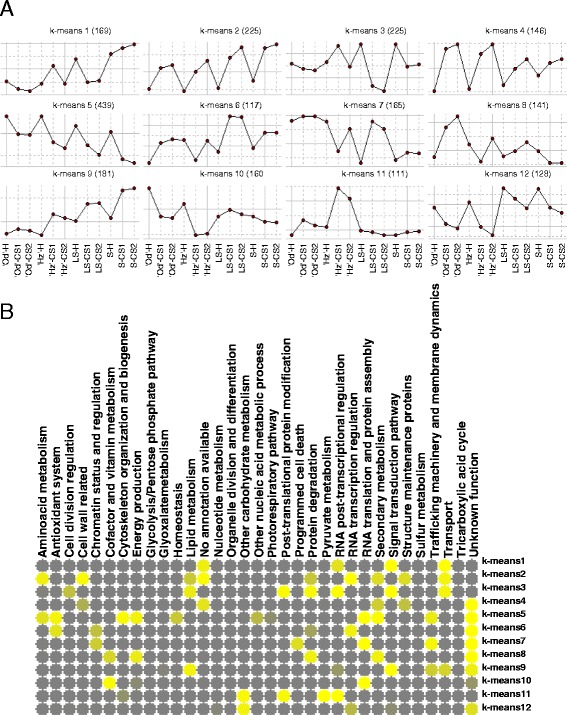
**Integrative analysis the transcriptomes of** **‘Oded’,** **‘Hermoza’** **and two pools of siblings from the Pop**-**DG population that cover a range of cold susceptibilities. A)** *K*-means clustering results of a set of 2207 genes with a 12-cluster limit. **B)** The functional categories overrepresented in each cluster are shown as a heatmap obtained with matrix2png. Enriched functional categories with Fisher test p-values < 0.05 are colored in grades of yellow. Tolerance-sensitivity range: Od > Hz > LS > S. H: Harvest; CS1: cold storage of 1 week at 5°C; CS2: cold storage of 2 weeks at 5°C; Od: ‘Oded’ peach; Hz: ‘Hermoza’ peach; LS: low sensitive Pop-DG pool; S: high sensitive Pop-DG pool.

To identify changes in gene expression that could be causally related to the tolerance/sensitivity to cold storage in peach fruit, we analyzed together the transcriptomes of Od, Hz and the LS and S pools by k-means clustering (Figure 6A; Additional file 6: Table S5). We reasoned that changes in gene expression common to all peach fruit are more likely to be part of core cold responses while differences may provide genes for the specific response of each fruit genotype to cold storage, and which may or may not be involved in tolerance. According to this, genes in clusters k-means 2, 5 and 9 (Figure 6A) were classified as part of the core cold response, but differ in their time/levels of expression and therefore are related to the degree of sensitivity/tolerance to cold. Given the common CI response that these fruit had was WLT; probably most of them were related to this disorder. Genes in cluster 2 and 9 were up-regulated by cold in a manner similar to their propensity to develop WLT (S > LS > Hz > Od; Figure 6A). The main difference between these clusters was that in k-means 9 the expression level at harvest correlated to sensitivity. Genes in cluster k-means 2 were enriched in *RNA transcription regulation*, *cell wall*, *transport*, *amino acid metabolism*, *secondary metabolism*, *structure maintenance proteins*, *lipid metabolism*, *protein degradation* and genes without any annotation (Figure 6B). Genes in cluster k-means 9 were enriched in *signal transduction pathway*, *lipid metabolism*, *unknown function*, *transport*, *trafficking machinery and membrane dynamics*, *RNA post*-*transcriptional regulation* (Figure 6B). In addition to up regulated genes, core cold responses also included down-regulated genes (cluster k-means 5). The genes in cluster k-means 5, enriched in *RNA translation and protein assembly*, *secondary metabolism*, *cytoskeleton organization and biogenesis*, *antioxidant system*, *energy production*, *trafficking machinery and membrane dynamics*, *aminoacid metabolism*, *homeostasis*, *other nucleic acid metabolic process* and genes with *unknown function*, were down-regulated by cold inversely to CI sensitivity (Figure 6B). Therefore, high levels of these genes contribute to the tolerance to cold storage.

The other clusters with interesting patterns included clusters k-means 1, 8 and 11. The genes in k-means 1, enriched in *signal transduction pathway*, *transport*, *RNA post*-*transcriptional regulation* and genes without any annotation available (Figure 6B) may be related to the higher sensitivity to WLT of the fruit in S pool. The genes in k-means 1 have expression levels at harvest that correlated to sensitivity degree and were up-regulated by cold in the S pool, but did not change in Od or were down regulated in Hz and in the LS pool (Figure 6B). The genes in the cluster k-means 11, enriched in *pyruvate metabolism*, *RNA post*-*transcriptional regulation*, *post*-*translational protein modification*, *other carbohydrate metabolism* and *cytoskeleton organization and biogenesis* (Figure 6B), were highly up-regulated by cold in Hz but unaffected in the three other fruits (Figure 6A). These genes are candidates regarding the sensitivity of Hz fruit to FB and FBL. The genes in cluster k-means 8 may be associated with the high tolerance of Od fruit to CI. They were up-regulated in Od by cold storage, but unchanged in the other fruits in comparison to Od (Figure 6A), and were enriched in *protein degradation*, *secondary metabolism*, *energy production*, *cofactor and vitamin metabolism* and genes with unknown function (Figure 6B).

In addition, and in order give more robustness to this comparison, we searched for the 50 genes that in our previous work [17] were validated in the contrasting pools and in 15 individual lines from the same population differing in the woolliness sensitivity by medium-throughput qRT-PCR. Forty of these genes were found in the comparison between Hz and Od and the pools (Additional file 7: Table S6). Out of them 34 were also confirmed in the 15 individual lines from the same population and 20 corresponded to the most relevant clusters (k-means 1, 2, 5 and 9). Overall, there was good agreement between the cluster analysis (Figure 6A) and the results for the validation in the individual lines. Out of the genes in cluster k-means 1, 2 and 9 (up-regulated by cold in a manner similar to their propensity to develop WLT), 15 out of 16 genes were found correlated to sensitivity in the individual Pop-DG lines. Similarly, five genes found in the cluster k-means 5 (down-regulated by cold in a manner similar to their propensity to develop WLT), were found associated to the high degree of tolerance of the individual lines. Further, genes such as *ACS1* (PPN004H06), *IAA27*/*PAP2* (PPN057F01), *glycosyltransferase* (PP1004E08) and an *unknown extracellular protein* (PP1001A01) validated in the comparison between Od and Hz (Additional file 3: Table S3) were found also validated in the individual lines (Figure 5, Additional file 7: Table S6 and [17]). Thus, it appears likely, that the genes identified in the comparison between Od, Hz and the pools play a role in the sensitivity/ tolerance of peach fruit to chilling injury.

### ROS-related transcriptomic signatures at harvest and during cold storage: ROSMETER analysis

**Figure 7:**
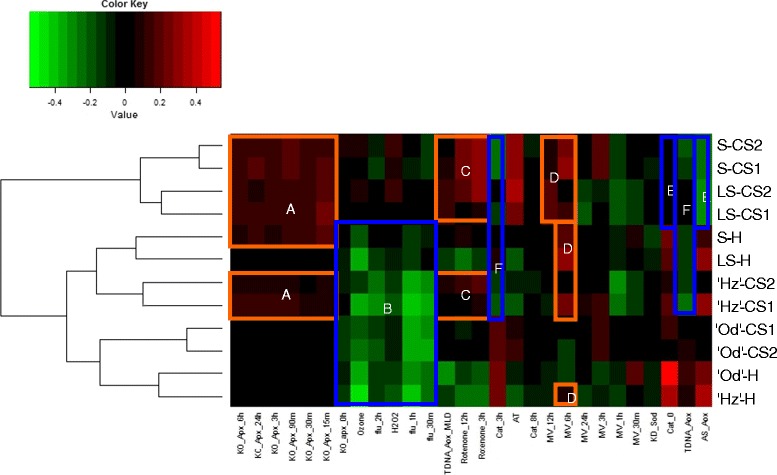
**ROSMETER analysis of the harvest and cold transcriptomes of** **‘Oded’,** **‘Hermoza’** **and LS and S peaches.** The ROS indices are listed on the abscissa and the Od, Hz, LS and S samples clustered by nearest neighbor correlation are shown on the ordinate. The color-coded results of correlations for each index are shown as a heat map. Correlation values are between 1 (complete positive correlation; red) and −1 (highest negative correlation; green), where 0 indicates no correlation (black). Correlation values above 0.12 and below −0.12 represent non-random correlations. ROS signatures discriminating fruits according to their sensitivity to CI are enclosed in bold boxes. Harvest; CS1: cold storage of 1 week at 5°C; CS2: cold storage of 2 weeks at 5°C.

A bioinformatic tool which was developed recently for Arabidopsis microarray data [18] to provide an organelle/type-dependent ROS-related transcriptomic signature was used to further characterize the differential peach responses to cold. ROSEMETER signatures were defined on the basis of transcriptome data obtained in experiments involving plant mutants in antioxidant enzymes or subjected to chemical applications that lead to increases in ROS production, thus providing information on the specificity of the transcriptomic response to oxidative stress. Since we had identified antioxidant system genes as differentially expressed at harvest and increasing in the resistant cultivar after one week at cold storage it was of interest to examine the ROS transcriptomic signature at harvest and during CS for the four fruit types (Figure 7). The ROSMETER analysis indicated that some signatures were capable of discriminating fruits according to their sensitivity to CI. The analysis revealed six distinct groups that clearly can be grouped according to the chilling sensitivity.

Group A includes all the knockout of cytoplasmic ascorbate peroxidase (KO-*APX1*) experiments, which are thought to represent cytoplasmic H_2_O_2_. These H_2_O_2_ indices correlated positively with sensitivity to CI before and during cold storage. In agreement a gene encoding a cytosolic ascorbate peroxidase (APX1; PPN071A07 Additional file 6: Table S5) was found among genes in cluster k-means 5 (Figure 6A), which may be related to a preformed mechanism of cold tolerance, since it is highest at harvest and in inverse relation to cold sensitivity.

Cluster B includes the indices of the conditional fluorescent (*flu*) mutant exposed to light 1 h, ozone and H_2_O_2_ treatments. All fruit at harvest showed negative correlations with these indices, but after cold storage, the most sensitive fruit (i.e., Pop-DG pools) showed positive correlations, being, in general, higher in pool S. This suggests that scavenging systems for apoplastic ROS and chloroplastic singlet oxygen could be active at harvest, but decrease during cold storage in parallel with sensitivity to CI.

Cluster C, which showed increase in Hz, LS and S fruit during storage, corresponds to rotenone treatments (3 and 12 h), an inhibitor of mitochondrial complex I, (i.e., NADH: ubiquinone oxidoreductase). Rotenone is associated with a mitochondrial stress but has not been shown to directly generate ROS [18]. In agreement, four of the 15 genes involved in energy production and enriching in k-means 5 (Figure 6) encode for NADH: ubiquinone oxidoreductase (Additional file 6: Table S5). Therefore low levels of mitochondrial complex I during cold storage could contribute to the sensitivity to cold.

Cluster D corresponds to 6 and 12 h methylviologen (MV) signatures, indicative of superoxide formation in the chloroplast and mitochondria [29]. 6hMV signature correlated positively with all fruits at harvest and cold stored, sensitive fruits (Hz, LS and S), while the most sensitive S and LS fruits also correlated positively with 12hMV signature, which may indicate secondary H_2_O_2_ stress effects [18].

Clusters E and F basically correlated negatively with sensitivity, included the indices of *CAT2 (0 and 3 h)*, alternative oxidase mutation (TDNA-*AOX1)*, and alternative oxidase antisense (AS-*AOX)*. The indices *CAT2*-0 and AS-*AOX* (cluster E) had a negative correlation with S and LS fruits during cold storage and a positive correlation with the degree of tolerance to CI, especially at harvest. The indices *CAT2*-3 h and TDNA-*AOX*1 (cluster F) correlated negatively with LS and S, both at harvest and during cold storage, and with Hz fruit during cold storage, which agree with data obtained from the direct comparison between Hz and Od (Figure 3; Additional file 3: Table S3). The fact that fruit tolerance is correlated positively with indices in cluster E and F, comprising mainly transcriptome data from mutants not exposed to any stress conditions, suggest tolerant fruit might have activated a compensatory scavenging mechanism.

These results indicate that although cultivars presented oxidative stress under cold storage, high levels of antioxidant activities in cytoplasm, mitochondria and chloroplast (chromoplast) are likely contribute to protection in the tolerant fruit.

## Discussion

### Integration of data from different peach genotypes and validation of the results

In this study an analysis of fruit transcript levels in response to CS in different peach genotypes is presented. In the first part of the of the study, the transcriptomes of Od and Hz at harvest and subjected to CS were analyzed using the Chillpeach microarray (Figures 2, 3 and 4). We validated the microarray data by qRT-PCR of ten genes (Additional file 5: Table S4), some of them reported previously to be associated to tolerance to WLT. We observed a high correlation between microarray and qRT-PCR data (Figure 5). The expression patterns of the single genes analyzed were in concordance (Figure 5B and Additional file 5: Table S4), although the level of expression was not confirmed for each gene in each sample. It is known from similar studies that the two technologies of expression analysis deliver qualitatively comparable data, however, the magnitude of such expression changes as reflected by microarray data tends to be generally compressed in comparison with qRT–PCR [16].

In the second part of the experiment, we performed a comparison of transcript levels between Od, Hz and two pools from the Pop-DG population (Figure 6 and Additional file 6: Table S5), recently analyzed at transcriptomic level [17]. A number of studies have reported changes in gene expression and protein activity in peach fruit in response to low temperature leading to CI (reviewed in [2]). However, differences in experimental approaches, genotypes, storage and shelf conditions (time and temperature) and also in the symptom assessment often result in lack of consistency of results [30-32]. In the case of microarray studies, the differences in technologies and cutoffs used for the identification of differentially expressed genes, the different genes represented on each array and technical differences in RNA and hybridization, analysis protocols and references used often hinder the identification of common regulated genes [33]. The expression changes identified in the two large experiments compared here, Od-Hz and S-LS pools, used the same sampling time, technical platform, RNA reference, analysis, protocols and p-values to identify differentially expressed genes, therefore overcoming this issue.

The peach lines in the Pop-DG populations used to produce LS and S pools were less tolerant to cold storage than Od and Hz. However, if the mechanism/program for tolerance was similar, our hypothesis was that genes highly expressed in the LS pool would show high expression levels in the tolerant Od compared to the sensitive counterpart, but the magnitude of the changes could be different. For the comparison we selected differentially expressed genes at one week of storage, the time where greatest differences in CI are observed. This removed considerable biological variation and added to the strength of the comparison. A criticism to our approach could be that we are setting a bias for the common cold regulated genes towards one of the CI symptoms, i.e., WLT.

We found close agreement between the significant changes detected by the two experiments (see Additional file 6: Table S5) and also with the sensitivity degree of individual lines of the Pop-DG population (see Additional file 7: Table S6). The low proportion of genes with opposite changes also supports this contention, and we feel that data from both experiments can be interpreted with confidence. Further, since the fruits of Od, Hz, LS and S pools cover a wide range in CI sensitivity, this comparison has allowed the identification of a set of genes with shared expression patterns (core cold response) that are candidates to be related to CI tolerance/sensitivity (Figure 6A, clusters k-means 2, 5 and 9) but also genotype specific responses (Figure 6A, clusters k-means 1, 8 and 11). Genes differentially expressed in one experiment but changing in opposite direction or not changing in the other could indicate a difference in the response due to the genotype or for other reasons [33].

However, although RNA expression data alone is insufficient for establishing a clear link between a gene/protein and the trait of interest, transcriptomics is an important first step to explore potential novel candidate genes for a particular process, which is the goal of this work. The data presented here, reinforce and extend previous reports, and provide insights into processes that are related to CI tolerance/sensitivity rather than simply being responses to cold.

### Quantitative differences in the subset of core cold responsive genes correlated with sensitivity to CI

Transcripts in the common cold regulated group showed expression values that correlated with sensitivity (Figure 6A). Furthermore, our results indicated that although reprogramming of the transcriptome underlies the core cold responses and the chilling sensitivity in peach fruit, many of these changes depend on the expression levels at harvest (Figure 6A). For the set of core cold responsive up-regulated genes, tolerant cultivars showed low expression levels both at harvest and during cold storage conditions, whereas sensitive cultivars showed increased expression in the cold (Figure 6A, cluster k-means 2 and 9). Interestingly, we could not identify a common core of cold response genes up-regulated in parallel with increased tolerance. This could be due to limitations of the Chillpeach microarray that was constructed with fruit from the Pop-DG mapping population [16], and which is less tolerant than Od. Alternatively, this may indicate that tolerant fruit were relatively less stressed at the cellular level compared to sensitive fruit and thus have a more limited response of the transcriptome, as has been described for salt and drought stressed rice [34].

### Expression of cell wall genes related to WLT at a pre-symptomatic stage

Alterations in cell wall related transcriptome, cell wall remodeling enzyme activities and in cell wall polymers metabolism in relation to WLT are normally detected during shelf life in cold sensitive cultivars but some have been reported to occur during extended cold storage [8,32]. Using Pop-DG siblings, a set of genes related to cell wall remodeling were found differentially expressed between S and LS pools, but no enrichment was found for this functional category [17]. In the current paper, we observed gene expression differences in cell wall genes during cold that could be associated to the eventual WLT phenotype that would develop in shelf life. It has been found that low levels of endo-PG activity combined with continuous activity of pectin methylesterase may lead to altered pectins during CS in fruit and this contributes to WLT when fruit are removed to SL [8,35]. Furthermore, endopolygalacturonase (endo-PG) was found in a quantitative trait loci (QTL) on linkage group LG4 for both FBL and WLT [36]. In agreement with this, we found a polygalacturonase inhibiting protein (PGIP), a pectin methyl esterase and pectin acetyl esterase among genes with low expression levels across all cultivars at harvest but which were up-regulated in parallel with increasing fruit sensitivity (k-means 2, Figure 6A; Table 2; Additional file 3: Table S3).

**Table 2:** **Genes discussed in the text correlated with sensitivity degree during cold storage** (**cluster k**-**means 2**)

**Function specific process**	**Chillpeach ID**	**Unigene annotation**	**Arab AGI**	**Arab gene symbol**	**Hormone signaling**	**Sugar signaling**/**partioning**	**Hormone and secondary metabolite biosynthesis regulation**	**Cell wall and cytoesqueleton related**	**Cell polarity**
**Aminoacid metabolism**									
**Alanine and Aspartate metabolism**	PPN065C10	Putative aspartate aminotransferase	AT1G80360	VAS1			Negative regulation of Trp-IAA and ET biosynthesis		
	PPN080E12	Putative aspartate aminotransferase	AT1G80360	VAS1			Negative regulation of Trp-IAA and ET biosynthesis		
**Cell wall related**									
**Cellulose biosynthesis**	PPN046D09	Cellulose synthase-like protein CslG	AT1G55850	CSLE1				SCW biosynthesis; hemicellulose biosynthesis	
**Hemicellulose biosynthesis**	PPN036E12	Glycosyltransferase	AT4G36890	IRX14				SCW biosynthesis;hemicellulose glucuronoxylan biosyntheis	
**Hemicellulose degradation**	PP1002E04	Alpha-L-arabinofuranosidase/beta-D-xylosidase	AT5G49360	BXL1				Pectin metabolism;trim b-xylan and a-arabinan side groups from the RG I.	
**Pectin degradation**	PPN041B11	Polygalacturonase-inhibiting protein	AT5G06860	PGIP1				Inhibition of degradation of the polygalacturonan	
	PPN047G10	Polygalacturonase-like protein	AT4G23500						
**Pectin methyl**-**esterification**	PP1004E01	Putative pectinesterase	AT2G26440						
	PPN001F02	Pectinacetylesterase family protein	AT5G23870						
	PPN066B05	Ripening-related protein-like	AT5G51520						
**UDP**-**L**-**arabinose**, **UDP**-**galacturonate and UDP**-**xylose Biosynthesis**	PPN062D06	UDP-arabinose 4-epimerase 1	AT1G30620	UXE1/MUR4		Sugar signaling		Arabionoglactan biosynthesis	
**Protein degradation**									
**Protease**	PP1004E07	Putative serine protease	AT5G67360	SBT1.7/ ARA12				Indirectly affects the pectin methylation status of mucilage and/or the primary CW	
	PPN009E02	Cysteine protease 14	AT4G35350	XCP1				SCW biosynthesis; postive regulation of thacheray element differentialion	
**RNA transcription regulation**									
**LUG**-**family**	PP1003C09	STY-L protein	AT2G32700	MUM1/ LUH				Control mucilage production and extrusion	
	PPN076D05	Transcriptional corepressor LEUNIG	AT4G32551	LUG	AUX signaling regulator			Control mucilage production and extrusion	
**NAC**-**family**	PPN054B06	No apical meristem protein-like	AT4G28500	anac073/ SND2				SCW biosynthesis; postive regulator of lignin, cellulose and hemicellulose biosyntehsis	
**WRKY**-**family**	PPN059A06	WRKY 13	AT2G37260	TTG2/WRKY44			Anthocyanin/PA polimerization regulation	mucilage production regulation	
**Secondary metabolism**									
**Anthocyanin metabolism**	PPN007E12	Anthocyanidin 3-O-glucosyltransferase	AT3G50740	UGT72E1				SCW biosynthesis; lignin biosynthesis	
**Carotenoid metabolism**	PP1005H08	Zeaxanthin epoxidase, chloroplast precursor	AT5G67030	ABA1/LOS6/ZEP	ABA biosynthesis			Mucilage production regulation	
**ET biosynthesis**	PPN004H06	1-aminocyclopropane-1-carboxylate synthase 1	AT3G61510	ACS1			ET biosynthesis		
**Phenylpropanoid metabolism**	PPN025B05	Cinnamoyl CoA reductase	AT1G15950	CCR1/IRX4				SCW biosynthesis; lignin biosythesis	
	PPN053B11	Cinnamyl alcohol dehydrogenase	AT4G37980	ELI3-1/CAD7				SCW biosynthesis; lignin biosythesis	
**Sterol metabolism**	PPN012F12	Delta(14)-sterol reductase	AT3G52940	FK/ HYD2	AUX and ET crosstalk; regulate AUX transporters localization in PM lipid microdomain formation and in the secretion machinery.			Cellulose, callose and lignin, VN development	Polar targeting of proteins to the PM;Lipid microdomains
	PPN063B12	Helix-turn-helix	AT4G37760	SQE3					
**Terpene metabolism**	PPN068G10	Beta-amyrin synthase	AT1G78950	BAS					
**Signal transduction pathway**									
**ABA signaling/** **Ca signal transducer**	PPN069F09	Putative serine/ threonine protein kinase PK11-C1	AT4G33950	OST1/ / SRK2E/ SNRK2-6	ABA	Sucrose metabolism regulation			
**ABA signaling/** **ABF phosphorylation**	PPN010B11	Serine-threonine protein kinase	AT1G78290	SNRK2.8/ SRK2C	ABA	sucrose signaling			
**Phosphorylation cascades/** **metabolic switch**	PPN054E02	AKIN beta3	AT2G28060	KINβ3	ABA	sucrose signaling			
**Trafficking machinery and membrane dynamics**									
**ER to Golgi**	PP1003D05	Root hair defective 3	AT3G13870	RHD3/ GOM8	AUX, ET			Required for CW biosynthesis and actin organization	Cell polarity regulation
**Sphingolipid metabolism**	PPN021D05	Similar to alkaline ceramidase	AT1G07380				Ceramide biosynthesis/ degradation		Polar targeting of proteins to the PM;Lipid microdomains
	PPN031D01	similar to alkaline ceramidase	AT1G07380				Ceramide biosynthesis/ degradation		Polar targeting of proteins to the PM;Lipid microdomains
**Transport**									
**AUX efflux to the apoplast**	PPN070B12	Multidrug resistance protein 11	AT3G28860	PGP19/ MDR11/ ABCB19	AUX transport				
**AUX transport into ER**	PP1004E09	Auxin Efflux Carrier family protein.	AT2G17500	PILS5	AUX transport				
	PPN075H08	Auxin Efflux Carrier family protein.	AT5G01990	PILS6	AUX transport				
**Carbohydrate transport**	PPN046B03	Sorbitol transporter	AT3G18830	PMT5/ PLT5		sugar partioning and homeostasis			
**Cooper transport**	PPN040A04	Copper transport protein-like	AT5G59040	COPT3					
**Ion transporter activity**	PPN016B02	Senescence-associated	AT2G17840	ERD7					
**Metal**-**ion transport**	PP1005G08	Metal tolerance protein C2	AT3G12100	MTP5					
	PPN007G12	Metal transporter Nramp3	AT2G23150	ATNRAMP3					
**Oligopepetide transport**	PPN029A02	Putative peptide transporter	AT3G01350						

*Abbreviations*: *AUX*: auxin; *ET*; ethylene; *ABA*: Abcisic acid; *PM*: plasma membrane; *CW*: cell wall; *SCW*: secondary cell wall; *ER*: endoplasmic reticulum; *MVB*/*LE*: microvesicular body/late endosome; *TGN*/*EE*: trans-golgy network/early endosome; *VSR*: vacuolar sorting receptors *VN*: vascular networks; *PA*: proanthocyanines; PIN; PIN formed auxin efflux carrier; *RG*:rhamnogalacturonan; *XyG*: xyloglucan.

References supporting information in Table 2 are provided in Additional file 8: Table S7.

During WLT development in shelf life pectin accumulation was observed in the intercellular spaces and inside parenchyma cells near to vascular bundles [37] and these modifications may begin during CS [8]. Moreover early histological studies indicate that during the last stages of peach fruit ripening a secretory system producing mucilage occurs within the mesocarp vascular bundles [38]. Our previous results have correlated BXL1 (β-xylosidase) and SBT1.7/ARA12 (serine protease) with WLT sensitivity in the Pop-DG population (Additional file 7: Table S6 and [17]). Current evidence suggests that these genes are required for the proper configuration of pectins in mucilage in seed and roots (see Table 2 and Additional file 8: Table S7 for references), and that there are analogies between fruit ripening and seed mucilage modification [39]. Here, we found these two genes also among the genes up-regulated by cold in a manner similar to fruit propensity to develop WLT (k-means 2; Figure 6A). Furthermore, among genes in cluster k-means 2 were also orthologs of other genes related to pectin configuration such as MUR4 (UDP-arabinose 4-epimerase) but also orthologs of genes required to control mucilage production and extrusion such as LEUNING (LUG), LUH/MUM1 (Leuning homolog), TTG2 (transparent testa glabra 2) and *LOS6*/*ABA1*, encoding a zeaxanthin epoxidase (Table 2). TTG2 and *LOS6*/*ABA1* regulate mucilage production [40,41] while, LUH/MUM1and LUG, function redundantly in promoting mucilage extrusion [42]. Thus it is likely that the changes in the expression of these genes are setting the stage for the WLT disorder in these pre-symptomatic fruit.

Cluster k-means 2 also includes genes related to non-cellulosic cell wall polysaccharide biosynthesis and lignification (Table 2) such as CSLE1 (cellulose synthase like 1), which was previously confirmed to be related to the sensitivity to WLT in individual lines of the Pop-DG population (Additional file 7: Table S6 and [17]) as well IRX14 (irregular xylem 14) CSLE1 (cellulose synthase like 1), IRX4/CCR4 (cinnamoyl Co-A reductase 4), UGT72E1 (UDP-glucosyltransferase 72E1), CAD7/ELI3-1 (cinnamyl alcohol dehydrogenase 7), XCP1 (X*YLEM CYSTEINE PEPTIDASE 1*) and SND2, a NAC domain protein that regulates the expression of lignin, cellulose and hemicellulose biosynthetic genes involved in secondary cell wall development in *Arabidopsis* fibers [43]. Thus, in addition to changes in pectin composition and biosynthesis, cold storage activates a secondary cell wall gene expression program in a WLT sensitivity dependence manner. In support of that, genes of cluster k-means 8 and cluster k-means 5 (increasing during CS in Od or associated to tolerance; Figure 6A and Table 3) include orthologs of negative regulators of lignin biosynthesis such as the myb-transcription factor MYB4 [44], WUSCHEL-related homeobox 13 (WOX13) [45], and two the MADS box genes, FRUTIFULL (FUL) and tomato AGAMOUS like TAGL1 (Table 3).

**Table 3:** **Genes discussed in the text correlated with tolerance**

**Function Specific process**		**Chillpeach ID**	**Unigene annotation**	**Arab AGI**	**Arab gene symbol**	**Hormone signaling**	**Sugar signaling/** **partioning**	**Hormone and secondary metabolite biosynthesis regulation**	**Cell wall and cytoesqueleton related**	**Cell polarity**
**k**-**means 5. Correlated with tolerance at harvest and during cold storage**										
**Aminoacid metabolism**										
	**Cyanide detoxification**	PPN075E10	Beta-cyanoalanine synthase 1	AT3G61440	CYSC1					
	**Methionine metabolism**	PPN034A06	1,2-dihydroxy-3-keto-5-methylthiopentene dioxygenase 4	AT5G43850	ARD4			Yang Cycle	associated to VN tissue	
		PPN034C12	1,2-dihydroxy-3-keto-5-methylthiopentene dioxygenase 3	AT4G14710	ARD2			Yang Cycle	associated to VN tissue	
		PPN072E05	Cystathionine gamma synthase	AT3G01120	MTO1/CGS1					
**Antioxidant system**										
	**GLUTHATHIONE**-**GLUTAREDOXIN AND THIOREDOXIN REDOX HOMEOSTASIS**	PPN039H11	Glutathione S-transferase	AT5G17220	TT19/GSTF12			PA monomer transporter		
**Cytoskeleton organization and biogenesis**										
	**Actin microfilament**-**actin depolimerization**	PPN047E05	Actin depolymerizing factor 2	AT5G59880	ADF3					
	**Microtubule**-**Microtubule binding and stabilization**	PPN073D05	Microtubule-associated proteins	AT5G55230	MAP65-1					
	**Microtubule**-**microtubule organization and formation**	PPN075E12	Tubulin folding cofactor B	AT3G10220	EMB2804/ TFC					
**RNA transcription regulation**										
	**AP2/** **EREBP family**	PPN054F05	AP2-related transcription factor	AT5G47220	ERF2	ET signaling			VN cell division	
	**AUX**/**IAA family**	PPN014H03	Auxin-induced protein AUX28	AT1G04250	AXR3/IAA17	AUX and ABA nuclear signaling; negative regulator				
		PPN057F01	AUX/IAA protein	AT4G29080	PAP2/IAA27	AUX nuclear signaling; negative regulator				
	**LIM**-**family**	PPN009B01	Pollen-specific protein SF3, putative	AT1G10200	WLIM1					
		PPN069C01	Transcription factor lim1	AT1G10200	WLIM1				Actin stabilizing protein	
	**MADS**-**box family**	PP1006G03	MADS-box transcription factor	AT5G60910	FUL/ AGL8			Positive regulatior of caroterne and anthocyanin biosynthesis,	Negative regulation of lignin	
		PPN042H02	MADS4	AT4G18960	AG	ET up-regulation		Postisitive carotene biosynthesis regulation;	Negative regulation of lignin biosynthesis	
**RNA translation and protein assembly**										
	**Regulation of protein biosynthesis**	PPN006H04	Translationally-controlled tumor protein homolog	AT3G16640	TCTP	AUX cytoplasmic signaling	Sugar signaling		CW biosynthesis regulation	
**Secondary metabolism**										
	**Anthocyanin metabolism**	PpLDOX (PpLDOX)	Leucoanthocyanidin dioxygenase		PpLDOX			Flavonoid/PA biosynthesis		
		PPN055C03	Anthocyanidin reductase	AT1G61720	BAN			PA biosynthess		
	**Aspartate biosyntheis**	PPN046D06	1-aminocyclopropane-1-carboxylate synthase	AT1G62960	ACS10					
	**Carotenoid metabolism**	PPN006A10	Phytoene synthase	AT5G17230	PSY					
		PPN067A01	Capsanthin/ capsorubin synthase	AT3G10230	LYC					
	**Cyanide detoxification**	PP1000E01	Cyanate hydratase	AT3G23490	CYN					
		PPN066B01	Nitrilase/cyanide hydratase and apolipoprotein N-acyltransferase family protein	AT5G12040						
	**Flavonoid metabolism**	PPN050G05	Dihydroflavonol 4-reductase-like	AT5G58490				Flavonoid/PA biosynthesis		
		PPN052H09	Chalcone synthase 2	AT5G13930	CHS/TT4	Negative regulation of AUX transport				
**Signal transduction pathway**										
	**Cytoplasmic TOR signaling**	PPN076G10	Protein lethal with sec thirteen 8-2	AT3G18140	LST8-1	AUX cytoplasmic signaling	Sugar signaling		CW biosynthesis regulation	
	**ET signaling/** **ET signal transduction**	PPN011G11	GTP-binding protein	AT3G46060	ARA3/RAB8A	ET signaling				
**Trafficking machinery and membrane dynamics**										
	**CME**;**EE**;**internalization and intracellular trafficking of PM proteins**	PPN011F03	Clathrin_L-chain	AT2G40060	CLC2	Regulates cellular AUX levels by controlling the abundance and distribution of PIN proteins at the PM				Cell polarity regulation
	**CME**;**internalization and intracellular trafficking of PM proteins**	PPN017G03	Calcium-binding EF-hand	AT3G01780	TPLATE	Regulates cellular AUX levels by controlling the abundance and distribution of PIN proteins at the PM			Regulation of cellulose synthesis by controlling the abundance of active CESA complexes at the PM	Cell polarity regulation
	**Endosomal sorting complex**	PPN060A04	Putative endosomal Vps protein complex subunit	AT5G22950	VPS24.1	Required for internalize PIN1, PIN2, and AUX1 to the MVB/ LE for vacuolar degradation				
	**Golgy to ER/** **COPI vesicles**	PPN044E10	ARF-like small GTPase 1	AT2G47170	ARF1A1C/BEX1	Essential for recycling of PIN transporters to the PM and for vacuolar targeting				Cell polarity
	**Retromer complex;LE to vacuole**	PPN007G03	Sorting nexin-like protein	AT5G06140	SNX1	Regulates both the recycling VSR from the TGN/ EE to the ER and the balance between vacuolar degradation and recycling of PIN proteins				
		PPN023B01	Ras-related protein Rab7	AT3G18820	RABG3F/RAB7B					
**k**-**means 8. Associated with high tolerance to chilling injury**										
**Aminoacid metabolism**										
	**AUX biosynthesis**	PPN058D11	Anthranilate synthase beta subunit	AT1G25220	ASB1	AUX biosynthesis				
**RNA transcription regulation**										
	**AUX/** **IAA family**	PP1009D02	IAA16 protein	AT1G04250	AXR3/IAA17	AUX and ABA nuclear signaling; negative regulator				
		PPN060G07	AUX/IAA protein	AT1G04240	IAA3/SHY2	AUX nuclear signaling; negative regulator				
	**HD**-**ZIP family**	PPN074H05	HB2 homeodomain protein	AT4G35550	HB-4/WOX13	AUX regulated			SCW biosynthesis; negative regulator lignin biosynthesis	
	**MYB**-**family**	PPN067A04	MYB-like DNA-binding domain protein	AT4G38620	MYB4				SCW biosynthesis; negative regulator lignin biosynthesis	
**Signal transduction pathway**										
	**ET signaling/** **ET receptor**	PPN054G06	Ethylene receptor	AT3G04580	EIN4	ET signaling				
**Transport**										
	**Cooper transport**	PPN035H02	Copper-transporting ATPase RAN1	AT5G44790	RAN1	ET signaling; delivers cooper ion into the ET receptors; is required for both ET binding and the receptor functionality				

*Abbreviations*: *AUX*:auxin; *ET*; ethylene; *ABA*: Abcisic acid; *PM*:plasma membrane; *CW*: cell wall; *SCW*: secondary cell wall; *ER*: endoplasmic reticulum; *MVB*/*LE*: microvesicular body/late endosome; *TGN*/*EE*:trans-golgy network/early endosome; *VSR*: vacuolar sorting receptors *VN*:vascular networks; *PA*: proanthocyanines; PIN; PIN formed auxin efflux carrier; *RG*: rhamnogalacturonan; *XyG*: xyloglucan.

References supporting information in Table 3 are provided in Additional file 8: Table S7.

### The maintenance of antioxidant systems and metabolites with antioxidant activity correlate with tolerance

Differences in expression of genes in the group of ‘down-regulated by cold’ could drive many of the responses to cold observed in peaches. These genes were constitutively expressed at high levels in the tolerant group of fruit and down regulated during cold storage in sensitive fruit, while in tolerant fruit they were less affected or even not changed (k-means 5 and 10; Figure 6). Previous studies have suggested that high constitutive gene expression prior to cold stress treatment might be part of a preformed tolerance mechanism in peach fruit [17,24], which may contribute to inhibition of some aspects of ripening and protect fruit during cold storage [17]. In particular, our results indicate that fruit with elevated levels at harvest and during cold storage of genes related to *protein biosynthesis*, especially ribosomal proteins, *energy production*, *antioxidant systems* and genes encoding for activities involved in the biosynthesis of secondary metabolites with antioxidant capacity such as carotenoids, flavonoids and proanthocyanins (k-means 5; Table 3; Additional file 3: Table S3 and Additional file 6: Table S5) were significantly less likely to develop CI. In agreement with these results, among genes correlated to WLT tolerance (cluster k-means 5 in Figure 6A; Table 3) there were the MADS box transcription factors AGAMOUS and FUL1, which have been described in other plants as positive regulators of carotenoid biosynthesis [46,47], flavonoids [46] and anthocyanins [48].

We previously reported that genes of the flavonoid and early proanthocyanin biosynthetic pathways such as chalcone synthase (CHS/TT4), leucoanthocyanidin dioxygenase (PpLDOX) and glutathione *S*-transferase 12 (GST12/TT19) were part of a preformed mechanism associated with cold tolerance [17,24,49]. The results here confirm these results (Table 3) and expand the list of genes related to these biosynthetic pathways to dihydroflavonol 4-reductase (DFR) and the ortholog of BANYUS (BAN), an anthocyanidin reductase (cluster k-means 5 in Figure 6A and Table 3). However, among genes in cluster k-means 2 (induced by cold in a sensitivity related manner) and k-means 1 (specific for high sensitivity to WLT) was the WRKY family transcription factor TTG2 (transparent testa glabra 2), which not only modulates mucilage production but also polymerization of proanthocyanidins [40] and AHA10, a putative P-type H^+^-ATPase involved in proanthocyanidin transport and polymerization (Tables 2 and 4). Interestingly, mutations in both, TTG2 and AHA10, increase the levels of proanthocyanidin monomers (i.e., catechin and epicatechin) [40,50]. Epicatechin showed negative correlation with chilling injury in peach fruit [51]. Taken together with this work, our results indicate that proanthocyanidin monomers may accumulate in tolerant fruit, while polymerized forms could be dominant in sensitive fruit.

**Table 4:** **Genes discussed in the text associated to high sensitivity to WLT and FB**

**Function specific process**	**Chillpeach ID**	**Unigene annotation**	**Arab AGI**	**Arab gene symbol**	**Hormone signaling**	**Sugar signaling/** **partioning**	**Hormone and secondary metabolite biosynthesis regulation**	**Cell wall and cytoesqueleton related**	**Cell polarity**
**k**-**means 1. Associated with high sensitivity to WLT**									
**Energy production**									
	**Plasma membrane ATP production**	PPN027C11	Plasma membrane proton ATPase	AT1G17260	AHA10			PA transport and polymerization	
**RNA transcription regulation**									
	**b**-**HLH family**	PPN080F10	Prf interactor 30137	AT2G27230	LHW	AUX signaling			VN establishment, maintenance, cell number and pattern
	**HB**-**family**	PPN069A12	BEL1-like homeodomain transcription factor	AT2G35940	BLH1				
**Signal transduction pathway**									
	**AUX signaling/** **AUX receptor E3 ubiquitin ligase SFC**-**TIR**	PPN078E01	Transport inhibitor response 1 protein	AT3G62980	TIR1	AUX nuclear signaling			
	**AUX signaling/** **Nuclear signaling pathway**	PPN078G01	Putative auxin-resistance protein	AT1G05180	AXR1	AUX nuclear signaling			
	**Calcium signaling/** **Calcium sensor**-**transducer**	PPN027B08	Calcium-dependent protein kinase	AT3G57530	CPK32	ABA			
	**Calcium signaling/** **Calcium signal transducer**	PPN013H01	Serine/ threonine kinase	AT5G58380	CIPK10/ SIP1/ SNRK3.8	ABA			
		PPN020F10	CBL-interacting protein kinase	AT4G30960	SNRK3.14/ CIPK6/ SIP3	ABA	sucrose signaling		
	**ET signaling**	PPN057C10	Ethylene signaling protein	AT5G03280	EIN2	ABA; positive regulator of ET signaling		Ethylene biosynthesis; positive regulator of ACS type I and negative regulator of ACS type II	VN cell division regulation
	**ET signaling/** **Culin E3 ubiquitin ligase**	PPN020G10	Ethylene-overproduction protein 1	AT3G51770	ETO1			repressor of ET biosynthesis (inhibits type II ACS)	VN cell division
	**Phosphorylation cascades/** **MAPK**	PPN020H02	Mitogen-activated protein kinase 4	AT4G01370	MPK4				Negative regulator of microtubule structure and stability; negative regulate MAP65-1
	**Phosphorylation cascades/** **metabolic switch**	PPN008G11	AKIN gamma	AT3G48530	KING1	ABA	Sucrose signaling		
	**Phosphorylation cascades/** **PP2A**	PPN037E11	Ser/thr protein phosphatase 2A regulatory subunit B’ gamma isoform	AT4G15415	ATB’GAMMA			Yang Cycle regulation	
**Transport**									
	**Carbohydrate transport**	PPN025D11	SLT1 protein	AT3G12570	FYD		Sugar partioning and homeostasis		
		PPN078G04	Putative membrane transporter	AT2G43330	INT1		Sugar partioning and homeostasis		
	**Cooper transport**	PPN025H09	Putative copper-transporting ATPase 3	AT1G63440	HMA5				
	**ion channel**	PPN023C11	Mechanosensitive ion channel	AT5G10490	MSL2				
	**Mg transport**	PPN001H12	MRS2-5	AT2G03620	MGT3				
	**oligopepetide transport**	PPN015D04	Metal-nicotianamine transporter YSL6	AT3G27020	YSL6				
		PPN028F10	Oligopeptide transporter OPT superfamily	AT5G55930	OPT1				
		PPN035B10	Oligopeptide transporter 7	AT4G10770	OPT7				
		PPN057F10	Oligopeptide transporter-like protein	AT3G54450					
**k**-**means 11. Associated with high sensitivity to FB**									
**Aminoacid metabolism**									
	**GABA biosynthesis**	PPN044B12	Glutamate decarboxylase, putative	AT3G17760	GAD5				
**Pyruvate and Reactive carbonyl species**									
	**Conversion of oxalacetate to PEP**	PP1002C02	Phosphoenolpyruvate carboxykinase [ATP]	AT4G37870	PEPCK;PCK1				
	**Pyruvate conversion to acetyl**-**CoA**	PPN054C12	Pyruvate dehydrogenase	AT1G59900	PDHE1-A				
		PPN059C05	Pyruvate dehydrogenase E1 beta subunit isoform 3	AT5G50850	MAB1/PDHE1-				
	**Pyruvate**-**lactate interconversions**	PP1006E06	Aldehyde dehydrogenase putative	AT1G44170	ALDH4/ALDH3H1	ABA			
		PPN035E06	Aldehyde dehydrogenase	AT1G44170	ALDH4/ALDH3H1	ABA			
		PPN038B05	Aldehyde dehydrogenase, putative	AT1G44170	ALDH4/ALDH3H1	ABA			

*Abbreviations*: *AUX*:auxin; *ET*; ethylene; *ABA*: Abcisic acid; *PM*:plasma membrane; *CW*: cell wall; *SCW*: secondary cell wall; *ER*: endoplasmic reticulum; *MVB*/*LE*:microvesicular body/late endosome; *TGN*/*EE*:trans-golgy network/early endosome; *VSR*:vacuolar sorting receptors *VN*: vascular networks; *PA*: proanthocyanines; PIN; PIN formed auxin efflux carrier; *RG*:rhamnogalacturonan; *XyG*:xyloglucan.

References supporting information in Table 4 are provided in Additional file 8: Table S7.

In addition, the ROSMETER results (Figure 7) suggest a genetic program for high levels of antioxidant activities in cytoplasm, mitochondria and chloroplast (chromoplast) in CI tolerant peach fruit, which correlated well with the expression of several genes of the antioxidant system or mitochondrial electron chain (particularly the ROS production site in mitochondria). Consistent with this, cold tolerance and cold acclimation have been associated with higher expression levels of antioxidant/scavenging systems, effective mitochondrial transport and protein synthesis in peach [17,24,52] and other plants [53-55]. In addition, ROSMETER results suggest tolerant fruit might have activated a compensatory scavenging mechanism [18]. Both direct comparison between Od and Hz and ROSMETER analysis highlight CAT2 as associated to the sensitivity to chilling (Additional file 3: Table S3; Figure 7). The reductive thiol pathways appear to compensate quite rapidly for catalase deficiency, leading to a new, more oxidized cellular redox state, notably reflected in adjustments of thiol-disulphide status [56]. In agreement, Od fruit had higher number and higher expression levels of genes related to gluthathione-glutaredoxin and thioredoxin redox homeostasis than the sensitive fruit (Additional file 3: Table S3) and the expression of these genes is correlated positively with tolerance (cluster k-means 5 and 8; Additional file 6: Table S5).

### A link between WLT at a pre-symptomatic stage and auxin responses and distribution

Our previous study [17] suggested that auxins play a role in the sensitivity/tolerance program induced by cold storage in peach fruit. We found that the expression of the of auxin transporters and positive regulators of nuclear auxin signaling correlated positively with the future WLT, while the expression of negative regulators of auxin signaling was associated with tolerance [17]. In support of this, clusters k-means 1, 2, and 9 (with higher levels in sensitive fruit; Figure 6A) include orthologs of plasma membrane and endoplasmic reticulum auxin efflux carriers (ABCB19/PGP19, PILS5 and PILS6; Table 2) as well nuclear signaling elements such as cullin CUL1/AXR6 and the auxin receptor TIR1/AXR1 (Tables 4 and Table 5). Also in agreement with our previous work, IAA/AUX proteins such as AXR3/IAA17 and IAA27 and SHY2/IAA3 (Table 3), encoding a negative regulators of auxin responses [57] were found in clusters k-means 5 and 8 (preformed tolerance and high-tolerance, respectively, Figure 6A). The expression of the ortholog of IAA27 is further supported by the qRT-PCR results (Figure 5B and Additional file 5: Table S4) and by the positive correlation of the ortholog of IAA27 with the degree of tolerance in individual lines from the Pop-DG population (Additional file 7: Table S6 and [17]).

**Table 5:** **Genes discussed in the text correlated with sensitivity at harvest and during cold storage** (**cluster k**-**means 9**)

**Function specific process**	**Chillpeach ID**	**Unigene annotation**	**Arab AGI**	**Arab gene symbol**	**Hormone signaling**	**Sugar signaling/** **partioning**	**Hormone and secondary metabolite biosynthesis regulation**	**Cell wall and cytoesqueleton related**	**Cell polarity**
**Cytoskeleton organization and biogenesis**									
**Microtubule stability and organization**	PPN077E06	Microtubule-associated protein	AT3G04630	WDL1				Negative regulator of microtubule structure and stability	
**Protein degradation**									
**Ubiquitin ligase E3 complex/** **SFC**-**culin**	PPN032H05	Cullin	AT4G02570	AXR6/CUL1	AUX nuclear signaling				
**RNA transcription regulation**									
**MYB**-**family**	PPN055C11	Sucrose responsive element binding protein	AT5G67300	MYBR1/MYB44	ABA, AUX, ET	Sucrose responsive element binding protein			
**Signal transduction pathway**									
**G**-**protein coupled receptor protein signaling pathway/** **G**-**protein complex**	PPN065B10	Guanine nucleotide binding protein (G-protein), alpha subunit family protein	AT1G31930	XLG3	ABA, AUX, ET	Sugar sensitivity			
**Phosphorylation cascades/** **MAPK**	PP1009F07	Trichoderma-induced protein kinase	AT3G45640	MPK3	Positive regulation of ACS type I		Ethylene biosynthesis; positive regulation of ACS type I	Pectin induced	
**Phosphorylation cascades/** **MAPKKK**	PPN071C11	protein kinase family protein/ankyrin repeat family protein	AT1G14000	VIK	AUX and BR signaling	Sugar partioning and homeostasis		VN formation	
**Phosphorylation cascades/** **PP2A**	PPN014G07	Serine/threonine-protein phosphatase 2A regulatory subunit A beta isoform	AT3G25800	PDF1/PP2AA2	Regulates PIN subcellular distribution				Cell polarity regulation
**Trafficking machinery and membrane dynamics**									
**CME**;**Vesicle coat/** **clathrin coated vesicles**	PP1003H08	Putative Clathrin coat assembly protein AP50	AT5G46630	AP2M	Regulates cellular AUX levels by controlling the abundance and distribution of PIN proteins at the PM			Regulates cellulose synthesis controlling the abundance and distribution of active CESA complexes at the PM	Cell polarity regulation
**Fatty acid biosyntheis**	PPN026B01	Carboxyl transferase alpha subunit	AT2G38040	CAC3			Fatty acid biosynthesis		
**Glycerolipid biosynthesis**	PPN008G03	Digalactosyldiacylglycerol synthase 1	AT3G11670	DGD1			Digalactosyl diacylglycerol biosynthesis		Polar targeting of proteins to the PM; Lipid microdomains
**Glycerolipid metabolism**	PPN065F12	phosphatidic acid phosphatase-related/PAP2-related	AT3G50920	LPPEPSILON1			Diacylglycerol biosynthesis		
**Phospholipid biosynthesis**	PPN008H07	Putative phospholipid cytidylyltransferase	AT2G38670	PECT1			Phosphoethanolamine biosynthesis		Polar targeting of proteins to the PM;Lipid microdomains
**Trans**-**Golgi network transport vesicle/** **COPI vesicles**	PPN002C04	ARF GTPase-activating domain-containing protein	AT5G13300	VAN3/SFC	required for either normal PIN1 cycling or for PID-directed efflux machinery relocation			Regulates formation of plant VN	Cell polarity regulation
**Transport**									
**Carbohydrate transport**	PP1003F09	Integral membrane protein,	AT1G75220	ERDL6		Sugar partioning and homeostasis			
**Cl**-**channel**	PPN078A03	Cl-channel, voltage gated; IMP dehydrogenase related 1	AT5G33280	CLCG					
**Na/** **K antiporter**	PPN064A01	Na+/ H+ antiporter	AT2G01980	SOS1					
**Nitrate transport**	PPN024D02	Nitrate transporter NRT1-2	AT1G18880	NRT1.9					
**Oligopepetide transport**	PPN005F03	Oligopeptide transporter 7	AT4G10770	OPT7					
	PPN064F08	POT family, putative	AT1G59740	NRT1/NPF4.3					
**Unknown transporter**	PPN066F09	Putative integral membrane protein	AT5G19980	GONST4		Sugar partioning and homeostasis		Is probably involved in the provision of GDP- sugars into the Golgi for CW polysaccharide synthesis such as RG-II and XyG	
**Unknown function**									
**Unknown interferon protein**	PPN065A05	Interferon-related developmental regulator family protein	AT1G27760	SAT32	ABA				
**Unkown Zinc finger RING**-**like**	PP1003D02	Ubiquitin ligase	AT3G23280	XBAT35	ET regulation ABA, AUX	Glucose			

*Abbreviations*: *AUX*:auxin; *ET*; ethylene; *ABA*: Abcisic acid; *PM*:plasma membrane;*CW*: cell wall; *SCW*: secondary cell wall; *ER*: endoplasmic reticulum; *MVB*/*LE*:microvesicular body/late endosome; *TGN*/*EE*:trans-golgy network/early endosome; *VSR*:vacuolar sorting receptors *VN*:vascular networks; *PA*: proanthocyanines; *PIN*; *PIN* formed auxin efflux carrier; *RG*:rhamnogalacturonan; *XyG*:xyloglucan.

References supporting information in Table 5 are provided in Additional file 8: Table S7.

The results here also highlight new auxin related genes as candidates to be involved in the tolerance/sensitivity to CS. The ortholog of anthranilate synthase (ASB1/WEI7), which is required for IAA synthesis (Table 3), was highly expressed in tolerant fruit in CS (k-means 8; Figure 6). Further, in cluster k-means 5 (high expression at harvest associated to tolerance, decreasing in storage; Table 3) are the translationally controlled tumour protein (TCTP) and LST8 (lethal with SEC13 protein 8), components of the TOR (target of rapamycin) signaling pathway, an integral part of the cytosolic auxin signaling pathway [58] that connects hormonal and nutrient pathways [59].

Taken together, the differential expression of several genes for auxin homeostasis, transport and signaling supports a strong connection between auxin metabolism and the CI tolerant/sensitive character of peach fruit. But how does auxin link with the expression changes observed for genes related to cell wall, antioxidants and other possible molecular signatures associated to WLT development at the pre-symptomatic stage? Evidence suggests that auxin can affect cell wall structure through both transcriptional, and non-transcriptional mechanisms, such the acidification-linked loosening of the wall (reviewed in [60]) and the TOR pathway [61]. We found that low levels of expression of TOR components were associated to sensitivity (cluster k-means 5, Figure 6A). Inhibition of TOR signaling caused specific changes to pectins and arabinogalactan protein components of cell walls [61]. However, via the cytoplasmic TOR pathway [59] auxin increases the overall cytoplasmic protein synthetic capacity of the cell [62]. This agrees with the higher levels of cell wall related genes in sensitive fruit and with the higher levels of genes related to protein biosynthesis in tolerant fruit. In addition, an important function of the TOR pathway is the regulation of mitochondrial activity and, hence, the production of ROS in animals [63] and in plants [61]. Thus, we suggest that while auxin changes are probably mainly related to cell wall in sensitive fruit, cytoplasmic auxin in tolerant fruit may be related to the maintenance of the translation machinery and the control of ROS.

### Ethylene is related to tolerance to cold storage

Ethylene reduction has been correlated with WLT sensitivity [27,28] and with the down regulation of some key cell wall activities associated to WLT development [8]. Zhou et al. [28] found that during prolonged cold storage, maintaining the ability of nectarine fruit to produce ethylene or adding exogenous ethylene to the storage atmosphere, prevented CI. Furthermore, correlating with ethylene production, the gene and protein expression of the ACO and ACS1were depleted during cold storage in fruit developing WLT during shelf life [27,28]. In agreement, we found that the most tolerant Od fruit have higher levels of both ACO and ACS (Additional file 3: Table S3). This is further supported by the qRT-PCR results (Figure 5B and Additional file 5: Table S4) and the positive correlation of the ACS1 with the tolerance degree in individual lines from the Pop-DG population (Additional file 7: Table S6 and [17]). Moreover, genes related to metabolism of the ethylene precursor methionine (salvage pathways and Yang cycle) and cyanide detoxification were in cluster k-means 5 (Table 3; Additional file 6: Table S5). It has been proposed that high rates of ethylene biosynthesis in climacteric fruit are supported by recycling of the ethylene precursor methionine via the Yang cycle [64] and by having an active system for handling cyanide, a byproduct of ethylene biosynthesis [65].

In addition several ethylene biosynthesis regulators and signaling elements were also differentially expressed between sensitive and tolerant fruit and their expression correlated with tolerance/sensitivity (Tables 2, 3, 4 and 5). EIN2 (ETHYLENE INSENSITIVE2) has been previously reported during cold storage in peach fruit [66] and it has been associated with cold sensitivity in Arabidopsis [67] and peach [17]. Both EIN2 and Ethylene-overproduction protein 1 (ETO1) (in cluster k-means 1; Figure 6A) are implicated in the negative regulation of type II ACS. EIN2 participates in the negative feedback regulation of ethylene biosynthesis by affecting the expression of ACS type II at transcriptional level [68], while ETO1 inhibits the enzymatic activity of type II ACS and targets it for 26S proteasome-mediated degradation [69]. In addition, in cluster k-means 2 (induced in CS and higher in sensitive fruit, Figure 6A) was VAS1 (reversal of vas3 phenotype; Table 2), recently identified as a cross-regulatory point controlling the flow through the auxin and ethylene biosynthetic pathways in response to shade [70]. VAS1 prevents over-accumulation of ethylene and auxin, thus preventing an exaggerated response to this environmental signal and *vas1* mutants accumulate ACC and auxins.

Furthermore, associated with high tolerance to cold (cluster k-means 8, Figure 6A) were the orthologs of the ethylene receptor EIN4 (ethylene insensitive 4) and RAN1, a P-type ATPase copper transporter that delivers the copper ion to the receptors and is required for both ethylene binding and the receptor functionality (Table 3). In Arabidopsis, EIN4 plays a positive role during cold acclimation in Arabidopsis [67], which coincides with their high expression in tolerant Od fruit (Additional file 6: Table S5). However, although EIN2 and EIN4 seem to play a similar role in cold acclimation in peach fruit and Arabidopsis, high levels of ethylene enhance tolerance to CI in peach while having a negative effect on Arabidopsis [67]. This difference may be explained by the different organs considered (fruits and leaves) and developmental processes. A lack of ethylene production during cold storage affects normal fruit ripening and leads to WLT [28].

### Sugar homeostasis and hormone crosstalk: auxin, ethylene, ABA

All three clusters (k-means 1, 2 and 9) associated to CI sensitivity were enriched in transport elements (Figure 6B). Besides the auxin transporters described above, these genes are rich in carbohydrate transporters and in oligopeptide/metal ion transporters (Tables 2, 4 and 5; Additional file 3: Table S3). This suggests that nutrient reallocation could play a role in the cell wall remodeling and metabolic changes happening in sensitive fruit. This may be the case of golgi nucleotide sugar transporter GONST4 (cluster k-means 9, Figure 5A), which is involved in the provision of GDP-fucose and GDP-l- galactose sugars into the Golgi for cell wall polysaccharide synthesis such as rhamnogalacturonan II and xyloglucan (Table 5), and ERDL6 (Early Responsive to Dehydration-Like 6) which functions as a vacuole glucose exporter (Table 5). Likewise, these transporters can also contribute to the sensitive character of peach fruit. Plants overexpressing ERDL6 or the sugar beet (*Beta vulgaris*) homolog B*v*IMP (Integral Membrane Protein) accumulated lower glucose and fructose in the vacuole than wild type and had reduced tolerance to cold [71].

In addition, the effect of cold on transporters can also reflect the stresses imposed to the fruit (cold, darkness and detachment), that may limit nutrient availability. It is described that in chilling sensitive peaches, glucose and fructose content increases during cold storage, while sucrose diminishes [72]. Emerging data indicate that sugar-derived signaling systems, including trehalose-6 phosphate (T6P), sucrose non-fermenting related kinase-1 (SnRK), and the TOR kinase complex also play important roles in regulating plant development through modulating nutrient and energy signaling and metabolic processes, especially under abiotic stresses where sugar availability is low (reviewed in [73]). Among signaling elements highly expressed in sensitive fruit were genes encoding for orthologs of several SnRKs of the three described groups SnRK1 (AKIN beta and aKING1, in clusters k-means 1 and 2), SnRK2 (SnRK2 OTS1/ SNRK2-6 and SNRK2.8, in cluster k-means 2) and SnRK3 (CIPK 10 and CIPK6; cluster k-means 1; Tables 2 and 4). Most of these genes have previously been associated to the chilling sensitive phenotype in peach [17]. Limited sucrose availability, osmotic stress and abscisic acid (ABA) activate the activity and the expression of SnRKs [74], which act as inhibitors of gene expression involved in different biosynthetic pathways [75]. The SnRK1 complex plays a central role in nutrient, darkness and stress [76]. Thus, it is likely that sucrose depletion by cold together with fruit detachment [72] enhances the expression of these genes. Furthermore, and in agreement with our results, the SnRK1 complex may play a role opposite to the one played by the TOR pathway in sensing energy [77] that promotes energy-consuming related cellular processes, such as mRNA translation when sucrose levels are high [77].

### Vesicle trafficking, membrane dynamics and cytoskeleton organization related to WLT at a pre-symptomatic stage

Our results indicate that differences in the expression levels of genes related to intracellular trafficking, cytoskeleton and lipid metabolism before and during cold storage (Tables 2, 3, 4 and 5 and Additional file 6: Table S5) could be related to the sensitivity or tolerance to CI in both a preformed (k-means 5; Figure 6A) and induced mechanism (k-means 2 and 9; Figure 6A). Similarly to other plants, this indicates that differences in membrane composition [78,79], cytoskeleton stability [80] and polar transport of proteins [81,82] participate in the response of peach fruit during cold. Differences in the expression of these genes could have a key role in the molecular phenotypes associated to the tolerance and sensitivity by regulating processes such as cell wall biosynthesis modifications and auxin distribution. Gonzalez Agüero et al. [83] suggested that alterations in the abundance of the endomembrane system components could have an important role in the development of WLT during cold and during shelf life by modifying the flow of polysaccharides and proteins to the cell wall. Furthermore, cytoskeleton [84,85] and lipid composition of membranes [86,87] are essential for, among other functions, polar distribution of membrane proteins, such as cell wall biosynthesis enzymes and auxin transporters.

### Gene expression related to sensitivity to FB and FBL at a pre-symptomatic stage

Hz was the only fruit that developed FBL and FB during the storage period. Although the analysis using the four fruit types is biased for WLT, the comparison of pools and Hz-Od experiments has identified a group of genes (k-means 11; Figure 6A) that only respond to cold in Hz fruit, and thus are good candidates to be related with Hz phenotype. This cluster is enriched in genes involved in the production of acetaldehyde and pyruvate metabolism (Figure 6B; Table 4). Further the same genes were identified as highly expressed in Hz compared to Od (Additional file 3: Table S3). Thus, acetaldehyde production could be related to the FBL and FB. FB is generally thought to be due to the action of polyphenol oxidase [9]. However, discoloration can also occur by non-enzymatic reactions through metal-polyphenol complexes [88]. This browning mechanism could be induced by chilling in Hz fruit. Hz fruit contained relatively high levels of expression of metal transport genes in comparison to Od and pools (Additional file 3: Tables S3 and Additional file 6: Table S5), which indicates a mobilization of metal ions associated to FB. Furthermore, high levels of PpLDOX correlated to BR sensitivity [36,49] and the results presented here indicate that Hz fruit have relatively high levels of expression of genes related to proanthocyanin monomer biosynthesis (Table 3). The combination of these two factors (i.e., high expression of both proanthocyanin and metal mobilization genes) with high expression of acetaldehyde production genes may increase the propensity of the fruit to FB when moved to shelf life. Lastly, among genes associated to the FB at a pre-symptomatic stage was an ortholog of glutamate decarboxylase (GAD5; Table 4). Glutamatade decarboxylase catalyzes the first and irreversible step of gamma aminobutyric acid (GABA; [89]). GABA has been shown to be a metabolic marker for core breakdown in pear [90]. These possible genes FB and FBL should be validated with additional cultivars.

## Conclusions

Our previous work prompted us to propose that in sensitive fruit a cold response program is activated, that is associated with dehydration/osmotic stress (induced by trapping water in cell wall pectins) and regulated by auxin distribution, ABA, and ethylene [17]. The results presented here indicate that sugar partitioning and demand during cold storage may also play an important role in the tolerance/sensitive mechanism and the interplay between sugar, cell wall and these three hormones (confirmed by the large list of regulators and signaling elements involved in the crosstalk of these factors, may have a role in the sensitive character even before fruit are cold stored. Furthermore, we validated and expanded the knowledge of the changes occurring in CI sensitive or tolerant fruit, by providing strong evidence of their correlation to sensitivity in a greater range of sensitivity/tolerance. This resulted in a variety of novel genes that provide targets on which further experimental analysis should focus. However, this is still a partial view and more actors may participate in the cold response to cold storage in peach fruit.

### Availability of supporting data

The data sets supporting the results of this article are available in the the ArrayExpress database (www.ebi.ac.uk/arrayexpress) under accession number E-MTAB-2708.

## Additional files

Additional file 1: Table S1. Gene-specific primers for qRT-PCR. Additional file 2: Table S2. Raw data. 3277 Chillpeach probes that met the threshold for hybridization quality. Expression data correspond to lowess M Log Ratio. Additional file 3: Table S3. Genes differentially expressed between ‘Oded’ and ‘Hermoza’ at harvest and during cold storage. In the Table there are the results for direct comparisons from the global analysis. The Table provides ID, the averaged lowess M Log Ratio and functional annotations. Additional file 4: Figure S1. Unsupervised two-dimensional hierarchical clustering of genes differentially expressed between ‘Oded’ and ‘Hermoza’ at harvest and during cold storage. Data represent averaged lowess M log ratio for three replicates. Color represents fold change (red: up-regulated and green: down-regulated). : Harvest; CS1: cold storage of 1 week at 5°C; CS2: cold storage of 2 weeks at 5°C; Od: ‘Oded’ peach; Hz: ‘Hermoza’ peach. Additional file 5: Table S4. RT-PCR gene expression values for representative genes and correlation with microarray data. Ten candidate genes were assayed by quantitative RT–PCR in fruits from Od and Hz at harvest and after 1 and 2 weeks of cold storage. For each gene this is shown Od and Hz values at harvest and the average gene expression pattern relative to harvest values in both expression platforms, microarray and qRT-PCR. The agreement between qRT-PCR and microarrays in expression profiles across samples is expressed as Pearson correlation coefficient. Additional file 6: Table S5. Comparison of genes differentially expressed at one week of cold storage between ‘Oded’ and Hermoza and between pools of siblings form the Pop-DG population. Data from fruits from the Pop-DG population cold stored were obtained from Pons et al. [17]. Additional file 7: Table S6. Comparison with genes previously validated in pools and in individual lines from the Pop-DG population. Data from fruits from the Pop-DG population cold stored were obtained from Pons et al. [17]. The cluster derived from Figure 6A is shown and the expression pattern at harvest and after one week of cold storage in pools LS and S. Also indicated are the genes validated in individual lines and pools. Additional file 8: Table S7. Genes and references supporting information in Tables 2, 3, 4 and 5.

## Abbreviations

CI: Chilling injury
FB: Flesh browning
FBL: Flesh reddening/bleeding
WLT: Woolliness/mealiness
Hz: Hermoza
Od: Oded
qRT-PCR: quantitative real-time PCR
ROS: Reactive oxygen species
M: Mature stage
SL: Shelf life
CS: Cold storage
S: high sensitive pool of siblings from the Pop-DG population
LS: Low sensitive pool of siblings from the Pop-DG population
