# Supplementary material for: Pre-symptomatic transcriptome changes during cold storage of chilling sensitive and resistant peach cultivars to elucidate chilling injury mechanisms
Source: BMC Genomics. 2015 Mar 26;16(1):245. doi: 10.1186/s12864-015-1395-6 (PMC4391166; doi:10.1186/s12864-015-1395-6)
Supplement: Additional file 4: Figure S1. — Unsupervised two-dimensional hierarchical clustering of genes differentially expressed between ‘Oded’ and ‘Hermoza’ at harvest and during cold storage. Data represent averaged lowess M log ratio for three replicates. Color represents fold change (red: up-regulated and green: down-regulated). : Harvest; CS1: cold storage of 1 week at 5°C; CS2: cold storage of 2 weeks at 5°C; Od: ‘Oded’ peach; Hz: ‘Hermoza’ peach. [file 12864_2015_1395_MOESM4_ESM.pdf]

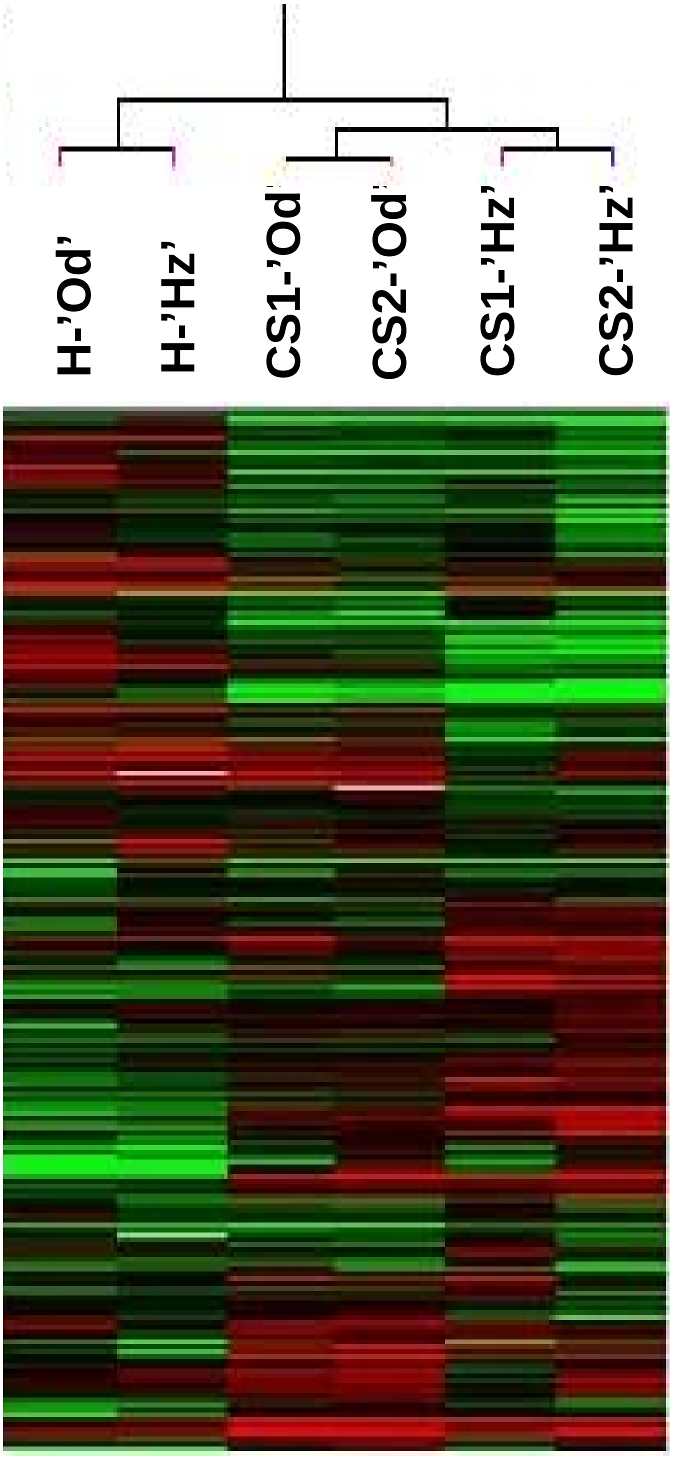

Figure S1. Unsupervised two-dimensional hierarchical clustering of genes differentially expressed between 'Oded' and 'Hermoza' at harvest and during cold storage. Data represent averaged lowess M log ratio for three replicates. Color represents fold change (red: up-regulated and green: down-regulated). : Harvest; CS1: cold storage of 1 week at 5ºC; CS2: cold storage of 2 weeks at 5ºC; 'Od': 'Oded' peach; 'Hz': 'Hermoza' peach
