# Supplementary material for: Pre-symptomatic transcriptome changes during cold storage of chilling sensitive and resistant peach cultivars to elucidate chilling injury mechanisms
Source: BMC Genomics. 2015 Mar 26;16(1):245. doi: 10.1186/s12864-015-1395-6 (PMC4391166; doi:10.1186/s12864-015-1395-6)
Supplement: Additional file 5: Table S4. — RT-PCR gene expression values for representative genes and correlation with microarray data. Ten candidate genes were assayed by quantitative RT–PCR in fruits from Od and Hz at harvest and after 1 and 2 weeks of cold storage. For each gene this is shown Od and Hz values at harvest and the average gene expression pattern relative to harvest values in both expression platforms, microarray and qRT-PCR. The agreement between qRT-PCR and microarrays in expression profiles across samples is expressed as Pearson correlation coefficient. [file 12864_2015_1395_MOESM5_ESM.doc]

Table S4. qRT-PCR validation of microarray data.

|  |  | **log2 qRT-PCR** | | | | | | **Microarray** | | | | | | **Pearson correlation**  **(R)** |
| --- | --- | --- | --- | --- | --- | --- | --- | --- | --- | --- | --- | --- | --- | --- |
|  | **Unigene annotation** | **Hz,H** | **Od,H** | **Hz, 1CS** | **Hz, 2CS** | **Od, 1CS** | **Od, 2CS** | **Hz,H** | **Od,H** | **Hz, 1CS** | **Hz, 2CS** | **Od, 1CS** | **Od, 2CS** |
| **Cell wall related** | | | | | | | | | | | | | | |
|  | **glycosyltransferase** | -1,346 | -1,583 | 1,353 | 1,966 | 1,859 | 2,489 | -1,529 | -1,685 | 0,551 | 1,028 | 0,897 | 1,677 | 0,998 |
|  | **Uknown extracellular protein/F17a17.37** | 0,923 | 1,063 | -1,102 | -2,510 | -0,960 | -1,883 | 1,761 | 2,231 | -1,474 | -2,096 | -2,015 | -2,434 | 0,943 |
|  | **β-Mannosidase** | 0,401 | 0,507 | 2,962 | 1,471 | 0,299 | 0,037 | 1,065 | 1,111 | 1,641 | 0,484 | 0,284 | -0,226 | 0,679 |
| **RNA transcription regulation** | | | | | | | | | | | | | | |
|  | **IAA27/PAP2** | 1,803 | 1,518 | -3,156 | -6,901 | -3,166 | -3,638 | 1,903 | 1,99 | -1,611 | -2,479 | -2,232 | -2,091 | 0,939 |
| **Secondary metabolism** | | | | | | | | | | | | | | |
|  | **ACO** | -0,668 | -0,871 | -0,491 | 0,915 | 1,610 | 1,307 | -1,757 | -1,949 | 0,676 | 1,141 | 1,054 | 1,542 | 0,830 |
|  | **ACC Synthase 1** | -5,108 | -7,361 | 1,180 | 3,244 | 6,391 | 8,686 | -2,513 | -2,815 | 0,222 | 1,335 | 1,172 | 3,458 | 0,975 |
|  | **Chalcone synthase 2** | 1,634 | -0,170 | -4,473 | -4,618 | 0,387 | -1,205 | -0,594 | -2,029 | -1,729 | -1,559 | 0,46 | 0,198 | 0,501 |
|  | **PpLDOX** | -0,356 | 0,576 | -0,206 | -1,543 | 0,842 | -0,371 | 0,169 | 0,288 | -0,159 | -0,253 | 0,106 | 0,218 | 0,657 |
| **Signal transduction pathway** | | | | | | | | | | | | | | |
|  | **Thaumatin-like protein 1** | -11,327 | -10,779 | 7,768 | 9,788 | 11,336 | 11,494 | -3,419 | -3,791 | 1,212 | 2,803 | 5,214 | 4,812 | 0,967 |
| **Trafficking machinery and membrane dynamics** | | | | | | | | | | | | | | |
|  | **Helix-hairpin-helix motif** | 0,409 | 0,638 | -0,897 | -1,070 | -1,064 | -0,742 | 0,426 | -0,641 | -1,279 | -1,87 | -0,913 | -0,513 | 0,701 |
