# Supplementary material for: Pre-symptomatic transcriptome changes during cold storage of chilling sensitive and resistant peach cultivars to elucidate chilling injury mechanisms
Source: BMC Genomics. 2015 Mar 26;16(1):245. doi: 10.1186/s12864-015-1395-6 (PMC4391166; doi:10.1186/s12864-015-1395-6)
Supplement: Additional file 8: Table S7. — Genes and references supporting information in Tables 2, 3, 4 and 5. [file 12864_2015_1395_MOESM8_ESM.pdf]

Table S4. Complete list of refereces for table 2

Function<br>Specific process	id	Unigene annotation	Arabidopsis AGI	Arabidopsis_thaliana_proteins most similar description	Arab Gene Name	Arab Gene Symbol	Hormone signaling	Sugar signaling/partioning	Hormone and secondary metabolite biosynthesis regulation	Cell wall and cytoesqueleton related	Cell polarity	References
**k-means9**												
**Cytoskeleton organization and biogenesis**												
**Microtubule stability and organization**	PPN077E06	Microtubule-associated protein	AT3G04630	protein with KLEEK protein-protein interaction domain	WVD2-LIKE 1	WDL1				Negative regulator of microtubule structure and stability		[1]
**Protein degradation**												
**Ubiquitin ligase E3 complex/SFC-culin**	PPN032H05	Cullin	AT4G02570	cullin family protein	AUXIN RESISTANT 6 ;CULLIN 1	AXR6/CUL1	AUX nuclear signaling					[2-4]
**RNA transcription regulation**												
**MYB-family**	PPN055C11	Sucrose responsive element binding protein	AT5G67300	myb family transcription factor	ARABIDOPSIS THALIANA MYB DOMAIN PROTEIN 44	MYBR1/MYB44	ABA, AUX, ET	Sucrose responsive element binding protein				[5-8]
**Signal transduction pathway**												
**G-protein coupled receptor protein signaling pathway/G-protein complex**	PPN065B10	Guanine nucleotide binding protein (G-protein), alpha subunit family protein	AT1G31930	extra-large guanine nucleotide binding protein, putative / G-protein	EXTRA-LARGE GTP-BINDING PROTEIN 3	XLG3	ABA, AUX, ET	sugar sensitivity				[9, 10]
**Phosphorylation cascades/MAPK**	PP1009F07	Trichoderma-induced protein kinase	AT3G45640	mitogen-activated protein kinase	MITOGEN-ACTIVATED PROTEIN KINASE 3	MPK3	positive regulation of ACS type I		positive regulation of ACS type I	pectin induced		[11-14]
**Phosphorylation cascades/MAPKKK**	PPN071C11	protein kinase family protein / ankyrin repeat family protein	AT1G14000	protein kinase family protein / ankyrin repeat family protein	VH1-INTERACTING KINASE	VIK	AUX and BR singaling	sugar partioning and homeostasis		vascular formation,		[15, 16]
**Phosphorylation cascades/PP2A**	PPN014G07	Serine/threonine-protein phosphatase 2A 65 kDa regulatory subunit A beta isoform	AT3G25800	serine/threonine protein phosphatase 2A 65 KDa regulatory subunit A	PROTEIN PHOSPHATASE 2A SUBUNIT A2	PDF1/PP2AA2	Regulates PIN subcellular distribution				cell polarity	[17, 18]
**Trafficking machinery and membrane dynamics**												
**CME;Vesicle coat/ clathrin coated vesicles**	PP1003H08	Putative Clathrin coat assembly protein AP50	AT5G46630	clathrin adaptor complexes medium subunit family protein	ADAPTOR PROTEIN-2 MU-ADAPTIN	AP2M	AUX signaling;regulates cellular auxin levels by controlling the abundance and distribution of PIN proteins at the plasma membrane			regulates cellulose synthesis controlling the abundance and distribution of active CESA complexes at the plasma membrane	Cell polarity regulation	[19, 20]
**Fatty acid biosyntheis**	PPN026B01	Carboxyl transferase alpha subunit	AT2G38040	acetyl co-enzyme A carboxylase carboxyltransferase alpha subunit family	ACETYL CO-ENZYME A CARBOXYLASE CARBOXYLTRANSFERAS E ALPHA SUBUNIT	CAC3			fatty acid biosynthesis			[21]
**Glycerolipid biosynthesis**	PPN008G03	Digalactosyldiacylglycerol synthase 1, chloroplast precursor	AT3G11670	digalactosyldiacylglycerol synthase 1	DIGALACTOSYL DIACYLGLYCEROL DEFICIENT 1	DGD1			digalactosyl diacylglycerol biosynthesis		polar targeting of proteins to the plasma membrane;Lipid microdomains	[22-25]
**Glycerolipid metabolism**	PPN065F12	phosphatidic acid phosphatase-related / PAP2-related	AT3G50920	phosphatidic acid phosphatase-related / PAP2-related	LIPID PHOSPHATE PHOSPHATASE EPSILON 1	LPPEPSILON1			diacylglycerol biosynthesis			[26]
**Phospholipid biosynthesis**	PPN008H07	Putative phospholipid cytidylyltransferase	AT2G38670	ethanolamine-phosphate cytidylyltransferase	PHOSPHORYLETHANOLA MINE CYTIDYLYLTRANSFERAS E 1	PECT1			phosphoethanolamine biosynthesis		polar targeting of proteins to the plasma membrane;Lipid microdomains	[23-25, 27]
**trans-Golgi network transport vesicle/ COPI vesicles**	PPN002C04	ARF GTPase-activating domain-containing protein	AT5G13300	ARF GTPase-activating domain-containing protein	VASCULAR NETWORK DEFECTIVE 3;SCARFACE	VAN3/SFC	AUX transport regulation;required for either normal PIN1 cycling or for PID-directed efflux machinery relocation			regulates formation of plant vascular networks	Cell polarity regulation	[28, 29]
**Transport**												
**carbohydrate transport**	PP1003F09	Integral membrane protein,	AT1G75220	vacuolar glucose exporter	ERD6-LIKE 6	ERDL6		sugar partioning and homeostasis				[30]

**Cl-channel**	PPN078A03	Cl-channel, voltage gated; IMP dehydrogenase related 1	AT5G33280	chloride channel-like protein	CHLORIDE CHANNEL G	CLCG					
**Na/K antiporter**	PPN064A01	Na+/H+ antiporter	AT2G01980	sodium proton exchanger,	SALT OVERLY SENSITIVE 1	SOS1					[31-33]
**nitrate transport**	PPN024D02	Nitrate transporter NRT1-2	AT1G18880	proton-dependent oligopeptide transport family protein	NITRATE TRANSPORTER 1.9	NRT1.9					
**oligopepetide transport**	PPN005F03	Oligopeptide transporter 7	AT4G10770	oligopeptide transporter OPT family protein	OLIGOPEPTIDE TRANSPORTER 7	OPT7					
	PPN064F08	POT family, putative	AT1G59740	proton-dependent oligopeptide transport (POT) family protein		NRT1/NPF4.3					
**Unknown transporter**	PPN066F09	Putative integral membrane protein	AT5G19980	Golgi-localized nucleotide-sugar transporter	GOLGI NUCLEOTIDE SUGAR TRANSPORTER 4	GONST4		sugar partioning and homeostasis		is probably involved in the provision of GDP-Fuc and GDP-l-Gal sugars into the Golgi for cell wall polysaccharide synthesis such as RG-II and XyG	[34-36]
**Unknown function**											
**Unknown interferon protein**	PPN065A05	interferon-related developmental regulator family protein	AT1G27760	interferon-related developmental regulator family protein	SALT-TOLERANCE 32	SAT32	ABA				[37]
**Unkown Zinc finger RING-like**	PP1003D02	ubiquitin ligase	AT3G23280	zinc finger (C3HC4-type RING finger) family protein / ankyrin repeat family protein	XB3 ORTHOLOG 5 IN ARABIDOPSIS THALIANA	XBAT35	ET regualtion,ABA, AUX	glucose			[38, 39]
**k-means2**											
**Aminoacid metabolism**											
**Alanine and Aspartate metabolism**	PPN065C10	Putative aspartate aminotransferase	AT1G80360	methionine-specific aminotransferase	REVERSAL OF SAV3 PHENOTYPE 1	VAS1			Negative regulation of Trp-IAA and ET biosynthesis		[40]
	PPN080E12	Putative aspartate aminotransferase	AT1G80360	methionine-specific aminotransferase	REVERSAL OF SAV3 PHENOTYPE 1	VAS1			Negative regulation of Trp-IAA and ET biosynthesis		[40]
**Cell wall related**											
**cellulose biosynthesis**	PPN046D09	Cellulose synthase-like protein CslG	AT1G55850	cellulose synthase family protein	CELLULOSE SYNTHASE LIKE E1	CSLE1				SCW biosynthesis;hemicellulose biosynthesis	[41]
**Hemicellulose biosynthesis**	PPN036E12	Glycosyltransferase	AT4G36890	glycosyl transferase family 43 protein	IRREGULAR XYLEM 14	IRX14				SCW biosynthesis;hemicellulose glucuronoxylan biosyntheis (SCW)	[42]
**Hemicellulose degradation**	PP1002E04	Alpha-L-arabinofuranosidase / beta-D-xylosidase	AT5G49360	bifunctional {beta}-D-xylosidase/{alpha}-L-arabinofuranosidase	BETA-XYLOSIDASE 1	BXL1				Pectin metabolism;trim b-xylan and a-arabinan side groups from the RG I.	[43]
**Pectin degradation**	PPN041B11	Polygalacturonase-inhibiting protein	AT5G06860	polygalacturonase inhibiting protein 1	POLYGALACTURONASE INHIBITING PROTEIN 1	PGIP1				inhibition of degradation of the polygalacturonan	
	PPN047G10	Polygalacturonase-like protein	AT4G23500	Pectin lyase-like superfamily protein							
**pectin methyl-esterification**	PP1004E01	Putative pectinesterase	AT2G26440	pectinesterase family protein,							
	PPN001F02	pectinacetylesterase family protein	AT5G23870	pectinacetylesterase family protein							
	PPN066B05	Ripening-related protein-like	AT5G51520	invertase/pectin methylesterase inhibitor family protein..							
**UDP-L-arabinose, UDP-galacturonate and UDP-xylose Biosynthesis**	PPN062D06	UDP-arabinose 4-epimerase 1	AT1G30620	NAD-dependent epimerase/dehydratase family protein	UDP-D-XYLOSE 4-EPIMERASE 1 ;MURUS 4	UXE1/MUR4		Sugar signaling		arabionoglactan biosynthesis	[44-47]
**Protein degradation**											

**protease**	PP1004E07	Putative serine protease	AT5G67360	cucumisin-like serine protease	subtilisin-like serine protease 1.7	SBT1.7/ARA12			indirectly affects the pectin methylation status of mucilage and/or the primary cell wall		[48]
	PPN009E02	Cysteine protease 14	AT4G35350	cysteine endopeptidase	XYLEM CYSTEINE PEPTIDASE 1	XCP1			SCW biosynthesis;postive regulation of thacheray element differentialion		[49]
**RNA transcription regulation**											
**LUG-family**	PP1003C09	STY-L protein	AT2G32700	WD-40 repeat family protein	MUCILAGE-MODIFIED 1 ;LEUNIG_HOMOLOG	MUM1/LUH			control mucilage production and extrusion		[50-52]
	PPN076D05	Transcriptional corepressor LEUNIG	AT4G32551	WD-40 repeat family protein (LEUNIG)	LEUNIG	LUG	AUX signaling regulator		control mucilage production and extrusion		[50-54].
**NAC-family**	PPN054B06	No apical meristem (NAM) protein-like	AT4G28500	no apical meristem (NAM) family protein	NAC DOMAIN CONTAINING PROTEIN 73 ;SECONDARY WALL-ASSOCIATED NAC DOMAIN PROTEIN 2	anac073/SND2			SCW biosynthesis;postive regulator of lignin, cellulose and hemicellulose biosyntehsis		[55, 56].
**WRKY-family**	PPN059A06	WRKY 13	AT2G37260	WRKY family transcription factor	TRANSPARENT TESTA GLABRA 2 ;WRKY family transcription factor 44	TTG2/WRKY44		anthocyanin /proanthocyanin polimerization regulation	mucilage production regulation,		[57]
**Secondary metabolism**											
**Anthocyanin metabolism**	PPN007E12	Anthocyanidin 3-O-glucosyltransferase	AT3G50740	UDPG:coniferyl alcohol glucosyltransferase	UDP-GLUCOSYL TRANSFERASE 72E1	UGT72E1			SCW biosynthesis;lignin biosynthesis		[58]
**Carotenoid metabolism**	PP1005H08	Zeaxanthin epoxidase, chloroplast precursor	AT5G67030	zeaxanthin epoxidase	ABA DEFICIENT 1 ;LOW EXPRESSION OF OSMOTIC STRESS-RESPONSIVE GENES 6 ;NON-PHOTOCHEMICAL QUENCHING 2 ;ARABIDOPSIS THALIANA ZEAXANTHIN EPOXIDASE ;ZEAXANTHIN EPOXIDASE ;IMPAIRED IN BABA-INDUCED STERILITY 3 ;ARABIDOPSIS THALIANA ABA DEFICIENT 1	ABA1/LOS6/ZEP	ABA biosynthesis		mucilage production regulation		[59, 60]
**ET biosynthesis**	PPN004H06	1-aminocyclopropane-1-carboxylate synthase 1	AT3G61510	1-aminocyclopropane-1-carboxylate synthase	ACC SYNTHASE 1	ACS1		ET biosynthesis			[61]
**Phenylpropanoid metabolism**	PPN025B05	Cinnamoyl CoA reductase	AT1G15950	cinnamoyl-CoA reductase	CINNAMOYL COA REDUCTASE 1 ; IRREGULAR XYLEM 4	CCR1/IRX4			SCW biosynthesis;lignin biosythesis		[62]
	PPN053B11	Cinnamyl alcohol dehydrogenase	AT4G37980	Cinnamyl alcohol dehydrogenase	ELICITOR-ACTIVATED GENE 3-1 ;CINNAMYL-ALCOHOL DEHYDROGENASE 7	ELI3-1/CAD7			SCW biosynthesis;lignin biosythesis		[63]
**Sterol metabolism**	PPN012F12	delta(14)-sterol reductase	AT3G52940	delta(14)-sterol reductase	FACKEL ; HYDRA 2	FK/HYD2	AUX and ET crosstalk;regulate auxin transporters localization in plasma membrane microdomains lipid microdomain formation and in the secretion machinery.		cellulose, callose and lignin, vascular development	polar targeting of proteins to the plasma membrane;Lipid microdomains	[64-67]
	PPN063B12	Helix-turn-helix, AraC type	AT4G37760	squalene monooxygenase	SQUALENE EPOXIDASE 3	SQE3					
**Terpene metabolism**	PPN068G10	Beta-amyrin synthase	AT1G78950	beta-amyrin synthase	BETA-AMYRIN SYNTHASE	BAS					

**Signal transduction pathway**												
**ABA signaling/ Ca signal transducer**	PPN069F09	Putative serine/threonine protein kinase PK11-C1	AT4G33950	similar to abscisic acid-activated protein kinase	OPEN STOMATA 1 ;SUCROSE NONFERMENTING 1-RELATED PROTEIN KINASE 2-6	OST1//SRK2E/SNRK2-6	ABA	Sucrose metabolism regulation				[68-71]
**ABA signaling/ABF phosphorylation**	PPN010B11	Serine-threonine protein kinase, putative	AT1G78290	serine/threonine protein kinase	SNF1-RELATED PROTEIN KINASE 2.8 ;SNF1-RELATED PROTEIN KINASE 2C	SNRK2.8/SRK2C	ABA	sucrose signaling				[72, 73]
**Phosphorylation cascades/metabolic switch**	PPN054E02	AKIN beta3	AT2G28060	5'-AMP-activated protein kinase beta-2 subunit protein		KINβ3	ABA	sucrose signaling				[72-75]
**Trafficking machinery and membrane dynamics**												
**ER to Golgi**	PP1003D05	Root hair defective 3	AT3G13870	root hair defective 3	GOLGI MUTANT 8 ;ROOT HAIR DEFECTIVE 3	RHD3/GOM8	AUX, ET			required for cell wall biosynthesis and actin organization	Cell polarity regulation	[76, 77]
**sphingolipid metabolism**	PPN021D05	similar to alkaline ceramidase	AT1G07380	ceramidase family protein					Ceramide biosynthesis/degradation		polar targeting of proteins to the plasma membrane;Lipid microdomains	[66, 67, 78, 79]
	PPN031D01	similar to alkaline ceramidase	AT1G07380	ceramidase family protein					Ceramide biosynthesis/degradation		polar targeting of proteins to the plasma membrane;Lipid microdomains	[66, 67, 78, 79]
**Transport**												
**AUX efflux to the apoplast**	PPN070B12	Multidrug resistance protein 11	AT3G28860	ATP-binding cassette (ABC) transporters	MULTIDRUG RESISTANCE PROTEIN 11 ;P-GLYCOPROTEIN 19 ATP-BINDING CASSETTE B19	PGP19/MDR11/ABCB19	AUX transport					[80-83]
**AUX transport into ER**	PP1004E09	Auxin Efflux Carrier family protein.	AT2G17500	Endoplasmic reticulum auxin efflux carrier family protein	PILS5	PILS5	AUX transport					[84, 85]
	PPN075H08	Auxin Efflux Carrier family protein.	AT5G01990	Endoplasmic reticulum auxin efflux carrier family protein	PILS6	PILS6	AUX transport					[84, 85]
**carbohydrate transport**	PPN046B03	Sorbitol transporter	AT3G18830	plasma membrane-localized polyol/cyclitol/monosaccharide-H+-symporter	ARABIDOPSIS THALIANA POLYOL/MONOSACCHARIDE TRANSPORTER 5 ;POLYOL/MONOSACCHARIDE TRANSPORTER 5 ;POLYOL TRANSPORTER 5	PMT5/PLT5		sugar partioning and homeostasis				
**Cooper transport**	PPN040A04	Copper transport protein-like	AT5G59040	copper transporter family protein	COPPER TRANSPORTER 3	COPT3						
**ion transporter activity**	PPN016B02	Senescence-associated	AT2G17840	senescence/dehydration-associated protein-related	EARLY-RESPONSIVE TO DEHYDRATION 7	ERD7						
**metal-ion transport**	PP1005G08	Metal tolerance protein C2	AT3G12100	cation efflux family protein / metal tolerance protein	METAL TRANSPORT PROTEIN 5	MTP5						
	PPN007G12	Metal transporter Nramp3	AT2G23150	NRAMP metal ion transporter 3	NATURAL RESISTANCE-ASSOCIATED MACROPHAGE PROTEIN 3	ATNRAMP3						
**oligopepetide transport**	PPN029A02	Putative peptide transporter	AT3G01350	proton-dependent oligopeptide transport family protein								
**k-means1**												
**Energy production**												
**plasma membrane ATP production**	PPN027C11	Plasma membrane proton ATPase	AT1G17260	proton-exporting ATPase 10	AUTOINHIBITED H-ATPASE ISOFORM 10	AHA10			proanthocyanidin transport and polymerization			[86, 87]
**RNA transcription regulation**												
**b-HLH family**	PPN080F10	Prf interactor 30137	AT2G27230	similar to basic helix-loop-helix(bHLH)-domain protein	LONESOME HIGHWAY	LHW	AUX signaling			Vascular establisment, maintenance, cell number and pattern		[88]
**HB-family**	PPN069A12	BEL1-like homeodomain transcription factor	AT2G35940	BEL1-like homeobox 4 protein	BEL1-LIKE HOMEODOMAIN 1	BLH1						[89, 90]
**Signal transduction pathway**												
**AUX signaling/ AUX receptor E3 ubiquitin ligase SFC-TIR**	PPN078E01	TRANSPORT INHIBITOR RESPONSE 1 protein	AT3G62980	E3 ubiquitin ligase SCF complex F-box subunit	TRANSPORT INHIBITOR RESPONSE 1	TIR1	AUX nuclear signaling					[2, 3]

**AUX signaling/Nuclear signaling pathway**	PPN078G01	Putative auxin-resistance protein	AT1G05180	auxin-resistance protein 1	AUXIN RESISTANT 1	AXR1	AUX nuclear signaling				[2-4]
**Calcium signaling/ Calcium sensor-transducer**	PPN027B08	Calcium-dependent protein kinase	AT3G57530	calcium-dependent protein kinase	CALCIUM-DEPENDENT PROTEIN KINASE 32	CPK32	ABA				[91]
**Calcium signaling/Calcium signal transducer**	PPN013H01	Serine/threonine kinase	AT5G58380	CBL-interacting protein kinase 10	CBL-INTERACTING PROTEIN KINASE 10 ;SNF1-RELATED PROTEIN KINASE 3.8 ;SOS3-INTERACTING PROTEIN 1 ;	CIPK10/SIP1/SNRK3.8	ABA				[92]
	PPN020F10	CBL-interacting protein kinase	AT4G30960	CBL-interacting protein kinase 6	CBL-INTERACTING PROTEIN KINASE 6 ;SOS3-INTERACTING PROTEIN 3 ;SNF1-RELATED PROTEIN KINASE 3.14	SNRK3.14/CIPK6/SIP3	ABA	sucrose signaling			[72, 73]
**ET signaling**	PPN057C10	Ethylene signaling protein	AT5G03280	ethylene-insensitive 2	ETHYLENE INSENSITIVE 2	EIN2	ABA;positive regulator of ET signaling		positive regulator of ACS type I and negative regulator of ACS type II	Vascular cel division regulation	[93-102]
**ET signaling/ Culin E3 ubiquitin ligase**	PPN020G10	Ethylene-overproduction protein 1	AT3G51770	tetratricopeptide repeat (TPR)-containing protein	ETHYLENE OVERPRODUCER 1	ETO1	repressor of ET biosynthesis (inhibits type II ACS)		repressor of ET biosynthesis (inhibits type II ACS)	vascular cell division	[102-104]
**Phosphorylation cascades/MAPK**	PPN020H02	Mitogen-activated protein kinase 4	AT4G01370	mitogen-activated protein kinase 4	MAP KINASE 4	MPK4				Negative regulator of microtubule structure and stability; negative regulate MAP65-1	[105]
**Phosphorylation cascades/metabolic switch**	PPN008G11	AKIN gamma	AT3G48530	CBS domain-containing protein	SNF1-RELATED PROTEIN KINASE REGULATORY SUBUNIT GAMMA	KING1	ABA	sucrose signaling			[72-75]
**Phosphorylation cascades/PP2A**	PPN037E11	Ser/thr protein phosphatase 2A regulatory subunit B' gamma isoform	AT4G15415	serine/threonine protein phosphatase 2A (PP2A) regulatory subunit B'gamma	B' regulatory subunit of PP2A	ATB'GAMMA			Yang Cycle regulation		[106]
**Transport**											
**carbohydrate transport**	PPN025D11	SLT1 protein	AT3G12570	HSP20-like chaperones superfamily protein	FYD	FYD		sugar partioning and homeostasis			
	PPN078G04	Putative membrane transporter	AT2G43330	Proton myo-inositol co-transporter	INOSITOL TRANSPORTER 1	INT1		sugar partioning and homeostasis			
**Cooper transport**	PPN025H09	Putative copper-transporting ATPase 3	AT1G63440	copper-exporting ATPase	HEAVY METAL ATPASE 5	HMA5					
**ion channel**	PPN023C11	MscS-Like mechanosensitive ion channel	AT5G10490	mechanosensitive ion channel domain-containing protein	MSCS-LIKE 2	MSL2					
**Mg transport**	PPN001H12	MRS2-5	AT2G03620	magnesium transporter CorA-like family protein	MAGNESIUM TRANSPORTER 3	MGT3					
**oligopepetide transport**	PPN015D04	Metal-nicotianamine transporter YSL6	AT3G27020	oligopeptide transporter OPT family protein	YELLOW STRIPE LIKE 6	YSL6					
	PPN028F10	Oligopeptide transporter OPT superfamily	AT5G55930	oligopeptide transporter OPT family protein	OLIGOPEPTIDE TRANSPORTER 1	OPT1					
	PPN035B10	Oligopeptide transporter 7	AT4G10770	oligopeptide transporter OPT family protein	OLIGOPEPTIDE TRANSPORTER 7	OPT7					
	PPN057F10	Oligopeptide transporter-like protein	AT3G54450	proton-dependent oligopeptide transport (POT) family protein							
**k-means11**											
**Aminoacid metabolism**											
**GABA biosynthesis**	PPN044B12	Glutamate decarboxylase, putative	AT3G17760	glutamate decarboxylase, putative, similar to glutamate decarboxylase GB:Q07346 (Petunia x hybrid...	glutamate decarboxylase 5 (GAD5),	GAD5					[107]
**Pyruvate and Reactive carbonyl species**											
**conversion of oxalacetate to PEP**	PP1002C02	Phosphoenolpyruvate carboxykinase [ATP]	AT4G37870	phosphoenolpyruvate carboxykinase (ATP), putative / PEP carboxykinase, putative / PEPCK, putative...	PHOSPHOENOLPYRUVATE CARBOXYKINASE 1 , PHOSPHOENOLPYRUVATE CARBOXYKINASE ,	PEPCK;PCK1					
**Pyruvate conversion to acetyl-CoA**	PPN054C12	Pyruvate dehydrogenase	AT1G59900	pyruvate dehydrogenase E1 component alpha subunit	PYRUVATE DEHYDROGENASE COMPLEX E1 ALPHA SUBUNIT ;PYRUVATE DEHYDROGENASE COMPLEX E1 ALPHA	PDHE1-A					

					SUBUNIT					
	PPN059C05	Pyruvate dehydrogenase E1 beta subunit isoform 3	AT5G50850	pyruvate dehydrogenase E1 component beta subunit	MACCI-BOU ,	MAB1/PDHE1-				
**pyruvate-lactate interconversions**	PP1006E06	Aldehyde dehydrogenase, putative	AT1G44170	aldehyde dehydrogenase	ALDEHYDE DEHYDROGENASE 3H1 , ALDEHYDE DEHYDROGENASE 4	ALDH4;ALDH3H1	ABA			[108]
	PPN035E06	Aldehyde dehydrogenase	AT1G44170	aldehyde dehydrogenase	ALDEHYDE DEHYDROGENASE 3H1 , ALDEHYDE DEHYDROGENASE 4	ALDH4;ALDH3H1	ABA			[108]
	PPN038B05	Aldehyde dehydrogenase, putative	AT1G44170	aldehyde dehydrogenase	ALDEHYDE DEHYDROGENASE 3H1 , ALDEHYDE DEHYDROGENASE 4	ALDH4;ALDH3H1	ABA			[108]
**k-means5**										
**Aminoacid metabolism**										
**cyanide detoxification**	PPN075E10	Beta-cyanoalanine synthase 1	AT3G61440	encodes a cysteine synthase isomer	CYSTEINE SYNTHASE C1	CYSC1				[109]
**methionine metabolism**	PPN034A06	1,2-dihydroxy-3-keto-5-methylthiopentene dioxygenase 4	AT5G43850	acireductone dioxygenase [iron(II)-requiring] activity	acireductone dioxygenase 4	ARD4		Yang Cycle	associated to vascular tissue	[110, 111]
	PPN034C12	1,2-dihydroxy-3-keto-5-methylthiopentene dioxygenase 3	AT4G14710	acireductone dioxygenase [iron(II)-requiring] activity	acireductone dioxygenase 2	ARD2		Yang Cycle	associated to vascular tissue	[110, 111]
	PPN072E05	Cystathionine gamma synthase	AT3G01120	cystathionine gamma-synthase, chloroplast / O-succinylhomoserine (Thiol)-lyase (CGS), identical t...	METHIONINE OVERACCUMULATION 1 (MTO1), CYSTATHIONINE GAMMA-SYNTHASE (CGS), A. THALIANA CYSTATHIONINE GAMMA-SYNTHASE 1 (ATCYS1), CYSTATHIONINE GAMMA-SYNTHASE 1 (CGS1),	MTO1;CGS1				[112]
**Antioxidant system**										
**GLUTHATHIONE-GLUTAREDOXIN AND THIOREDOXIN REDOX HOMEOSTASIS**	PPN039H11	Glutathione S-transferase	AT5G17220	glutathione S-transferase	TRANSPARENT TESTA 19 ;GLUTATHIONE S-TRANSFERASE PHI 12	TT19/GSTF12		proanthocyanin monomer transporter		[113, 114]
**Cytoskeleton organization and biogenesis**										
**actin microfilament-actin depolimerization**	PPN047E05	Actin depolymerizing factor 2	AT5G59880	actin-depolymerizing factor 3	ACTIN DEPOLYMERIZING FACTOR 3	ADF3				[115, 116]
**Microtubule-Microtubule binding and stabilization**	PPN073D05	MICROTUBULE-ASSOCIATED PROTEINS	AT5G55230	MICROTUBULE-ASSOCIATED PROTEINS	MICROTUBULE-ASSOCIATED PROTEINS 65-1	MAP65-1				[117, 118]
**Microtubule-microtubule organization and formation**	PPN075E12	Tubulin folding cofactor B	AT3G10220	tubulin folding cofactor B	EMBRYO DEFECTIVE 2804	EMB2804/TFC				[119]
**RNA transcription regulation**										
**AP2/EREBP family**	PPN054F05	AP2-related transcription factor	AT5G47220	ethylene response factor subfamily B-3 of ERF/AP2 transcription factor	ETHYLENE RESPONSIVE ELEMENT BINDING FACTOR 2	ERF2	ET signaling		vascular cell division	[102, 120]
**AUX/IAA family**	PPN014H03	Auxin-induced protein AUX28	AT1G04250	auxin-responsive protein / indoleacetic acid-induced protein 17	INDOLE-3-ACETIC ACID INDUCIBLE 17 ;AUXIN RESISTANT 3	AXR3/IAA17	AUX and ABA nuclear signaling; negative regulator			[121]
	PPN057F01	AUX/IAA protein	AT4G29080	auxin-responsive protein / indoleacetic acid-induced protein 27	INDOLE-3-ACETIC ACID INDUCIBLE 27 ;PHYTOCHROME-ASSOCIATED PROTEIN 2	PAP2/IAA27	AUX nuclear signaling;negative regulator			[122, 123]
**LIM-family**	PPN009B01	Pollen-specific protein SF3, putative	AT1G10200	transcription factor LIM, putative	widely expressed LIM protein 1	WLIM1				[124]
	PPN069C01	Transcription factor lim1	AT1G10200	transcription factor LIM, putative	widely expressed LIM protein 1	WLIM1		Actin stabilizing protein	Actin stabilizing protein	[124]
**MADS-box family**	PP1006G03	MADS-box transcription factor	AT5G60910	agamous-like MADS box protein AGL8 / FRUITFULL	FRUITFULL ;AGAMOUS-LIKE 8	FUL/AGL8		positive regulatior of caroterne and anthocyanin biosynthesis,	negative regulation of lignin	[125-128]
	PPN042H02	MADS4	AT4G18960	floral homeotic protein AGAMOUS (AG), contains an ACG start codon	AGAMOUS	AG	ET up-regulation	postisitive carotene biosynthesis regulation;	negative regulation of lignin	[129-131]

				(Riechmann, Ito, and Meyerowitz...						biosynthesis		
**RNA translation and protein assembly**												
**regulation of protein biosynthesis**	PPN006H04	Translationally-controlled tumor protein homolog	AT3G16640	translationally controlled tumor family protein	TRANSLATIONALLY CONTROLLED TUMOR PROTEIN	TCTP	AUX cytoplasmic signaling	Sugar signaling		Cell wall biosynthesis regulation		[132, 133]
**Secondary metabolism**												
**Anthocyanin metabolism**	PpLDOX (PpLDOX)	leucoanthocyanidin dioxygenase				PpLDOX			flavonoid/proanthocyanin biosynthesis			[134]
	PPN055C03	Anthocyanidin reductase	AT1G61720	dihydroflavonol 4-reductase	BANYULS	BAN			proanthocyanin biosynthess			[134]
**Aspartate biosyntheis**	PPN046D06	1-aminocyclopropane-1-carboxylate synthase	AT1G62960	aminotransferase with broad specificity for aspartate and aromatic amino acids	ACC SYNTHASE 10	ACS10						[135]
**Carotenoid metabolism**	PPN006A10	Phytoene synthase	AT5G17230	phytoene synthase	PHYTOENE SYNTHASE	PSY						
	PPN067A01	Capsanthin/capsorubin synthase, chloroplast precursor	AT3G10230	lycopene beta cyclase	LYCOPENE CYCLASE	LYC						
**cyanide detoxification**	PP1000E01	Cyanate hydratase	AT3G23490	cyanate lyase family	CYANASE	CYN						[136]
	PPN066B01	Nitrilase/cyanide hydratase and apolipoprotein N-acyltransferase family protein;	AT5G12040	Nitrilase/cyanide hydratase and apolipoprotein N-acyltransferase family protein								[109, 137]
**Flavonoid metabolism**	PPN050G05	Dihydroflavonol 4-reductase-like	AT5G58490	cinnamoyl-CoA reductase family					flavonoid/proanthocyanin biosynthesis			[138]
	PPN052H09	Chalcone synthase 2	AT5G13930	naringenin-chalcone synthase	CHALCONE SYNTHASE ;TRANSPAREN T TESTA 4	CHS/TT4	negative regulation of auxin transport					[139, 140]
**Signal transduction pathway**												
**Cytoplasmic TOR signaling**	PPN076G10	protein LETHAL WITH SEC THIRTEEN 8-2	AT3G18140	transducin family protein / WD-40 repeat family protein	LETHAL WITH SEC THIRTEEN 8-1	LST8-1	AUX cytoplasmic signaling	Sugar signaling		Cell wall biosynthesis regulation		[133, 141]
**ET signaling/ET signal transduction**	PPN011G11	GTP-binding protein	AT3G46060	Ras-related protein/ small GTP-binding protein	RAB GTPASE HOMOLOG 8A	ARA3/RAB8A	ET signaling					[142]
**Trafficking machinery and membrane dynamics**												
**CME;Early endosome;internalization and intracellular trafficking of PM proteins**	PPN011F03	Clathrin_L-chain	AT2G40060	clathrin light chain	CLATHRIN LIGHT CHAIN 2	CLC2	AUX signaling;regulates cellular auxin levels by controlling the abundance and distribution of PIN proteins at the plasma membrane				Cell polarity regulation	[143]
**CME;internalization and intracellular trafficking of PM proteins**	PPN017G03	Calcium-binding EF-hand	AT3G01780	cytokinesis adaptor protein targeted to the cell plate		TPLATE	AUX signaling;regulates cellular auxin levels by controlling the abundance and distribution of PIN proteins at the plasma membrane			regulation of cellulose synthesis by controlling the abundance of active CESA complexes at the plasma membrane	Cell polarity regulation	[144, 145]
**Endosomal sorting complex**	PPN060A04	Putative endosomal Vps protein complex subunit	AT5G22950	SNF7 family protein	Vacuolar protein-sorting-associated protein 24	VPS24.1	required for internalize PIN1, PIN2, and AUX1 to the MVB/late for vacuolar degradation					[146]
**Golgy to ER/ COPI vesicles**	PPN044E10	ARF-like small GTPase 1	AT2G47170	ADP-ribosylation factor 1	ADP-ribosylation factor 1 AC1/bfa-visualized exocytic trafficking defective1	ARF1A1C/BEX1	Essential for recycling of PIN auxin transporters to the plasma membrane and for vacuolar targeting				cell polarity	[147]
**Retromer complex;Late endosome to vacuole**	PPN007G03	Sorting nexin-like protein	AT5G06140	phox (PX) domain-containing protein	SORTING NEXIN 1	SNX1	Regulates both the recycling vacuolar sorting receptors (VSR) from the TGN/EE to the ER and the balance between vacuolar degradation and recycling of PIN					[148, 149]

							proteins		
	PPN023B01	Ras-related protein Rab7	AT3G18820	Ras-related GTP-binding protein	RAB GTPASE HOMOLOG G3F	RABG3F/RAB7B			[150]
**k-means8**									
**Aminoacid metabolism**									
**AUX biosynthesis**	PPN058D11	Anthranilate synthase beta subunit	AT1G25220	anthranilate synthase beta subunit	ANTHRANILATE SYNTHASE BETA SUBUNIT 1	ASB1	AUX biosynthesis		[151]
**RNA transcription regulation**									
**AUX/IAA family**	PP1009D02	IAA16 protein	AT1G04250	auxin-responsive protein / indoleacetic acid-induced protein 17	INDOLE-3-ACETIC ACID INDUCIBLE 17 ;AUXIN RESISTANT 3	AXR3/IAA17	AUX and ABA nuclear signaling; negative regulator		[121]
	PPN060G07	AUX/IAA protein	AT1G04240	auxin-responsive protein / indoleacetic acid-induced protein 3	INDOLE-3-ACETIC ACID INDUCIBLE 3;SHORT HYPOCOTYL 2	IAA3/SHY2	AUX nuclear signaling;negative regulator		[152]
**HD-ZIP family**	PPN074H05	HB2 homeodomain protein	AT4G35550	homeobox-leucine zipper protein (HB-2) / HD-ZIP protein	WUSCHEL RELATED HOMEOBOX 13	HB-4/WOX13	AUX regulated	SCW biosynthesis;negative regulator lignin biosynthesis	[153, 154]
**MYB-family**	PPN067A04	MYB-like DNA-binding domain protein	AT4G38620	myb family transcription factor	MYB DOMAIN PROTEIN 4	MYB4		SCW biosynthesis;negative regulator lignin biosynthesis	[155]
**Signal transduction pathway**									
**ET signaling/ET receptor**	PPN054G06	Ethylene receptor	AT3G04580	Ethylene receptor, subfamily 2.	ETHYLENE INSENSITIVE 4	EIN4	ET signaling		[99, 156, 157]
**Transport**									
**Cooper transport**	PPN035H02	Copper-transporting ATPase RAN1	AT5G44790	copper-exporting ATPase / responsive-to-antagonist 1	RESPONSIVE-TO-ANTAGONIST 1 ;	RAN1	ET signaling; delivers cooper ion into the ET receptors;is required for both ET binding and the receptor functionality		[158]

Abreviatures: AUX:auxin; ET; ethylene; ABA: Abcisic acid; PM:plasma membrane;CW: cell wall; SCW: secondary cell wall; ER: endoplasmic reticulum; MVB/ LE:microvesicular body/ late endosome; TGN/ EE:trans-golgy network/ early endosome; VSR:vacuolar sorting receptors VN:vascular networks; PA: proanthocyanines; PIN; PIN formed auxin efflux carrier; RG:rhamnogalacturonan; XyG:xyloglucan

References

1. Perrin RM, Wang Y, Yuen CYL, Will J, Masson PH: **WVD2 is a novel microtubule-associated protein in Arabidopsis thaliana**. *The Plant Journal* 2007, **49**(6):961-971.
2. Dharmasiri N, Dharmasiri S, Estelle M: **The F-box protein TIR1 is an auxin receptor**. *Nature* 2005, **435**(7041):441-445.
3. Gray WM, Kepinski S, Rouse D, Leyser O, Estelle M: **Auxin regulates SCFTIR1-dependent degradation of AUX/ IAA proteins**. *Nature* 2001, **414**(6861):271-276.
4. Kepinski S, Leyser O: **The Arabidopsis F-box protein TIR1 is an auxin receptor**. *Nature* 2005, **435**(7041):446-451.
5. Lee B, Henderson D, Zhu J: **The Arabidopsis cold-responsive transcriptome and its regulation by ICE1**. *Plant Cell* 2005, **17**(11):3155 - 3175.
6. Kranz HD, Denekamp M, Greco R, Jin H, Leyva A, Meissner RC, Petroni K, Urzainqui A, Bevan M, Martin C *et al*: **Towards functional characterisation of the members of theR2R3-MYBgene family fromArabidopsis thaliana**. *The Plant Journal* 1998, **16**(2):263-276.
7. Jung C, Seo JS, Han SW, Koo YJ, Kim CH, Song SI, Nahm BH, Choi YD, Cheong J-J: **Overexpression of AtMYB44 Enhances Stomatal Closure to Confer Abiotic Stress Tolerance in Transgenic Arabidopsis**. *Plant Physiology* 2008, **146**(2):623-635.

8. Liu R, Chen L, Jia Z, Lü B, Shi H, Shao W, Dong H: **Transcription Factor AtMYB44 Regulates Induced Expression of the ETHYLENE INSENSITIVE2 Gene in Arabidopsis Responding to a Harpin Protein**. *Molecular Plant-Microbe Interactions* 2010, **24**(3):377-389.
9. Ding L, Pandey S, Assmann SM: **Arabidopsis extra-large G proteins (XLGs) regulate root morphogenesis**. *Plant J* 2008, **53**(2):248-263.
10. Pandey S, Monshausen GB, Ding L, Assmann SM: **Regulation of root-wave response by extra large and conventional G proteins in Arabidopsis thaliana**. *The Plant Journal* 2008, **55**(2):311-322.
11. Moscatiello R, Mariani P, Sanders D, Maathuis FJM: **Transcriptional analysis of calcium-dependent and calcium-independent signalling pathways induced by oligogalacturonides**. *Journal of Experimental Botany* 2006, **57**(11):2847-2865.
12. Taj G, Agarwal P, Grant M, Kumar A: **MAPK machinery in plants: Recognition and response to different stresses through multiple signal transduction pathways**. *Plant Signaling & Behavior* 2010, **5**(11):1370-1378.
13. Han L, Li G-J, Yang K-Y, Mao G, Wang R, Liu Y, Zhang S: **Mitogen-activated protein kinase 3 and 6 regulate Botrytis cinerea-induced ethylene production in Arabidopsis**. *The Plant Journal* 2010, **64**(1):114-127.
14. Li G, Meng X, Wang R, Mao G, Han L, Liu Y, Zhang S: **Dual-Level Regulation of ACC Synthase Activity by MPK3/ MPK6 Cascade and Its Downstream WRKY Transcription Factor during Ethylene Induction in Arabidopsis**. *PLoS Genet* 2012, **8**(6):e1002767.
15. Ceserani T, Trofka A, Gandotra N, Nelson T: **VH1/ BRL2 receptor-like kinase interacts with vascular-specific adaptor proteins VIT and VIK to influence leaf venation**. *The Plant Journal* 2009, **57**(6):1000-1014.
16. Wingenter K, Trentmann O, Winschuh I, Hörmiller II, Heyer AG, Reinders J, Schulz A, Geiger D, Hedrich R, Neuhaus HE: **A member of the mitogen-activated protein 3-kinase family is involved in the regulation of plant vacuolar glucose uptake**. *The Plant Journal* 2011, **68**(5):890-900.
17. Dai M, Zhang C, Kania U, Chen F, Xue Q, Mccray T, Li G, Qin G, Wakeley M, Terzaghi W *et al*: **A PP6-Type Phosphatase Holoenzyme Directly Regulates PIN Phosphorylation and Auxin Efflux in Arabidopsis**. *The Plant Cell Online* 2012, **24**(6):2497-2514.
18. Michniewicz M, Zago MK, Abas L, Weijers D, Schweighofer A, Meskiene I, Heisler MG, Ohno C, Zhang J, Huang F *et al*: **Antagonistic Regulation of PIN Phosphorylation by PP2A and PINOID Directs Auxin Flux**. *Cell* 2007, **130**(6):1044-1056.
19. Bashline L, Li S, Anderson CT, Lei L, Gu Y: **The Endocytosis of Cellulose Synthase in Arabidopsis Is Dependent on μ2, a Clathrin-Mediated Endocytosis Adaptin**. *Plant Physiology* 2013, **163**(1):150-160.
20. Kim SY, Xu Z-Y, Song K, Kim DH, Kang H, Reichardt I, Sohn EJ, Friml J, Juergens G, Hwang I: **Adaptor Protein Complex 2–Mediated Endocytosis Is Crucial for Male Reproductive Organ Development in Arabidopsis**. *The Plant Cell Online* 2013.
21. Tang F, Moore Jr TS: **Enzymes of the Primary Phosphatidylethanolamine Biosynthetic Pathway in Postgermination Castor Bean Endosperm (Developmental Profiles and Partial Purification of the Mitochondrial CTP:Ethanolaminephosphate Cytidylyltransferase)**. *Plant Physiology* 1997, **115**(4):1589-1597.
22. Klaus D, Härtel H, Fitzpatrick LM, Froehlich JE, Hubert J, Benning C, Dörmann P: **Digalactosyldiacylglycerol Synthesis in Chloroplasts of the Arabidopsis dgd1 Mutant**. *Plant Physiology* 2002, **128**(3):885-895.
23. Zhang C, Tian S: **Peach fruit acquired tolerance to low temperature stress by accumulation of linolenic acid and N-acylphosphatidylethanolamine in plasma membrane**. *Food Chemistry* 2010, **120**(3):864-872.
24. Degenkolbe T, Giavalisco P, Zuther E, Seiwert B, Hincha DK, Willmitzer L: **Differential remodeling of the lipidome during cold acclimation in natural accessions of Arabidopsis thaliana**. *The Plant Journal* 2012, **72**(6):972-982.
25. Szymanski J, Brotman Y, Willmitzer L, Cuadros-Inostroza Á: **Linking Gene Expression and Membrane Lipid Composition of Arabidopsis**. *The Plant Cell Online* 2014, **26**(3):915-928.
26. Nakamura Y, Tsuchiya M, Ohta H: **Plastidic Phosphatidic Acid Phosphatases Identified in a Distinct Subfamily of Lipid Phosphate Phosphatases with Prokaryotic Origin**. *Journal of Biological Chemistry* 2007, **282**(39):29013-29021.

27. Mizoi J, Nakamura M, Nishida I: **A Study of the Physiological Function of Phosphatidylethanolamine in Arabidopsis**. In: *Advanced Research on Plant Lipids*. Edited by Murata N, Yamada M, Nishida I, Okuyama H, Sekiya J, Hajime W: Springer Netherlands; 2003: 377-380.
28. Sieburth LE, Muday GK, King EJ, Benton G, Kim S, Metcalf KE, Meyers L, Seamen E, Van Norman JM: **SCARFACE Encodes an ARF-GAP That Is Required for Normal Auxin Efflux and Vein Patterning in Arabidopsis**. *The Plant Cell Online* 2006, **18**(6):1396-1411.
29. Koizumi K, Naramoto S, Sawa S, Yahara N, Ueda T, Nakano A, Sugiyama M, Fukuda H: **VAN3 ARF–GAP-mediated vesicle transport is involved in leaf vascular network formation**. *Development* 2005, **132**(7):1699-1711.
30. Poschet G, Hannich B, Raab S, Jungkunz I, Klemens PAW, Krueger S, Neuhaus E, Büttner M: **A Novel Arabidopsis Vacuolar Glucose Exporter is involved in cellular Sugar Homeostasis and affects Composition of Seed Storage Compounds**. *Plant Physiology* 2011.
31. Shi H, Ishitani M, Kim C, Zhu J-K: **The Arabidopsis thaliana salt tolerance gene SOS1 encodes a putative Na+/ H+ antiporter**. *Proceedings of the National Academy of Sciences* 2000, **97**(12):6896-6901.
32. Shi H, Quintero FJ, Pardo JM, Zhu J-K: **The Putative Plasma Membrane Na+/ H+ Antiporter SOS1 Controls Long-Distance Na+ Transport in Plants**. *The Plant Cell Online* 2002, **14**(2):465-477.
33. Quintero FJ, Ohta M, Shi H, Zhu J-K, Pardo JM: **Reconstitution in yeast of the Arabidopsis SOS signaling pathway for Na+ homeostasis**. *Proceedings of the National Academy of Sciences* 2002, **99**(13):9061-9066.
34. Handford MG, Sicilia F, Brandizzi F, Chung JH, Dupree P: **Arabidopsis thaliana expresses multiple Golgi-localised nucleotide-sugar transporters related to GONST1**. *Molecular Genetics and Genomics* 2004, **272**(4):397-410.
35. Reyes F, Orellana A: **Golgi transporters: opening the gate to cell wall polysaccharide biosynthesis**. *Current Opinion in Plant Biology* 2008, **11**(3):244-251.
36. Wulff C, Norambuena L, Orellana A: **GDP-Fucose Uptake into the Golgi Apparatus during Xyloglucan Biosynthesis Requires the Activity of a Transporter-Like Protein Other Than the UDP-Glucose Transporter**. *Plant Physiology* 2000, **122**(3):867-878.
37. Park MY, Chung MS, Koh HS, Lee DJ, Ahn SJ, Kim CS: **Isolation and functional characterization of the Arabidopsis salt-tolerance 32 (AtSAT32) gene associated with salt tolerance and ABA signaling**. *Physiol Plant* 2009, **135**(4):426-435.
38. Carvalho SD, Saraiva R, Maia TM, Abreu IA, Duque P: **XBAT35, a Novel Arabidopsis RING E3 Ligase Exhibiting Dual Targeting of Its Splice Isoforms, Is Involved in Ethylene-Mediated Regulation of Apical Hook Curvature**. *Molecular Plant* 2012, **5**(6):1295-1309.
39. Yuan X, Zhang S, Liu S, Yu M, Su H, Shu H, Li X: **Global Analysis of Ankyrin Repeat Domain C3HC4-Type RING Finger Gene Family in Plants**. *PLoS One* 2013, **8**(3):e58003.
40. Zheng Z, Guo Y, Novák O, Dai X, Zhao Y, Ljung K, Noel JP, Chory J: **Coordination of auxin and ethylene biosynthesis by the aminotransferase VAS1**. *Nat Chem Biol* 2013, **9**(4):244-246.
41. Liepman AH, Wilkerson CG, Keegstra K: **Expression of cellulose synthase-like (Csl) genes in insect cells reveals that CslA family members encode mannan synthases**. *Proceedings of the National Academy of Sciences of the United States of America* 2005, **102**(6):2221-2226.
42. Lee C, Teng Q, Huang W, Zhong R, Ye Z-H: **The Arabidopsis Family GT43 Glycosyltransferases Form Two Functionally Nonredundant Groups Essential for the Elongation of Glucuronoxylan Backbone**. *Plant Physiology* 2010, **153**(2):526-541.
43. Arsovski AA, Popma TM, Haughn GW, Carpita NC, McCann MC, Western TL: **AtBXL1 Encodes a Bifunctional β-d-Xylosidase/ α-l-Arabinofuranosidase Required for Pectic Arabinan Modification in Arabidopsis Mucilage Secretory Cells**. *Plant Physiology* 2009, **150**(3):1219-1234.
44. Reiter W-D, Chapple C, Somerville CR: **Mutants of Arabidopsis thaliana with altered cell wall polysaccharide composition**. *The Plant Journal* 1997, **12**(2):335-345.
45. Burget EG, Reiter W-D: **The mur4 Mutant of Arabidopsis Is Partially Defective in the de Novo Synthesis of Uridine Diphosphol-Arabinose**. *Plant Physiology* 1999, **121**(2):383-390.

46. Burget EG, Verma R, Mølhøj M, Reiter W-D: **The Biosynthesis of l-Arabinose in Plants: Molecular Cloning and Characterization of a Golgi-Localized UDP-d-Xylose 4-Epimerase Encoded by the MUR4 Gene of Arabidopsis**. *The Plant Cell Online* 2003, **15**(2):523-531.
47. Li Y, Smith C, Corke F, Zheng L, Merali Z, Ryden P, Derbyshire P, Waldron K, Bevan MW: **Signaling from an Altered Cell Wall to the Nucleus Mediates Sugar-Responsive Growth and Development in Arabidopsis thaliana**. *The Plant Cell Online* 2007, **19**(8):2500-2515.
48. Rautengarten C, Usadel B, Neumetzler L, Hartmann J, Büssis D, Altmann T: **A subtilisin-like serine protease essential for mucilage release from Arabidopsis seed coats**. *The Plant Journal* 2008, **54**(3):466-480.
49. Funk V, Kositsup B, Zhao C, Beers EP: **The Arabidopsis Xylem Peptidase XCP1 Is a Tracheary Element Vacuolar Protein That May Be a Papain Ortholog**. *Plant Physiology* 2002, **128**(1):84-94.
50. Huang J, DeBowles D, Esfandiari E, Dean G, Carpita NC, Haughn GW: **The Arabidopsis Transcription Factor LUH/ MUM1 Is Required for Extrusion of Seed Coat Mucilage**. *Plant Physiology* 2011, **156**(2):491-502.
51. Bui M, Lim N, Sijacic P, Liu Z: **LEUNIG_HOMOLOG and LEUNIG Regulate Seed Mucilage Extrusion in ArabidopsisF**. *Journal of Integrative Plant Biology* 2011, **53**(5):399-408.
52. Walker M, Tehseen M, Doblin MS, Pettolino FA, Wilson S, Bacic T, Golz JF: **The transcriptional regulator LEUNIG_HOMOLOG regulates mucilage release from the Arabidopsis testa**. *Plant Physiology* 2011.
53. Navarro C, Efremova N, Golz JF, Rubiera R, Kuckenberg M, Castillo R, Tietz O, Saedler H, Schwarz-Sommer Z: **Molecular and genetic interactions between STYLOSA and GRAMINIFOLIA in the control of Antirrhinum vegetative and reproductive development**. *Development* 2004, **131**(15):3649-3659.
54. Gonzalez D, Bowen AJ, Carroll TS, Conlan RS: **The Transcription Corepressor LEUNIG Interacts with the Histone Deacetylase HDA19 and Mediator Components MED14 (SWP) and CDK8 (HEN3) To Repress Transcription**. *Molecular and Cellular Biology* 2007, **27**(15):5306-5315.
55. Zhong R, Lee C, Zhou J, McCarthy RL, Ye Z-H: **A Battery of Transcription Factors Involved in the Regulation of Secondary Cell Wall Biosynthesis in Arabidopsis**. *The Plant Cell Online* 2008, **20**(10):2763-2782.
56. Hussey S, Mizrachi E, Spokevicius A, Bossinger G, Berger D, Myburg A: **SND2, a NAC transcription factor gene, regulates genes involved in secondary cell wall development in Arabidopsis fibres and increases fibre cell area in Eucalyptus**. *BMC Plant Biology* 2011, **11**(1):173.
57. Johnson CS, Kolevski B, Smyth DR: **TRANSPARENT TESTA GLABRA2, a Trichome and Seed Coat Development Gene of Arabidopsis, Encodes a WRKY Transcription Factor**. *The Plant Cell Online* 2002, **14**(6):1359-1375.
58. Lim E-K, Jackson RG, Bowles DJ: **Identification and characterisation of Arabidopsis glycosyltransferases capable of glucosylating coniferyl aldehyde and sinapyl aldehyde**. *FEBS Letters* 2005, **579**(13):2802-2806.
59. Marin E, Nussaume L, Quesada A, Gonneau M, Sotta B, Hugueney P, Frey A, Marion-Poll A: **Molecular identification of zeaxanthin epoxidase of Nicotiana plumbaginifolia, a gene involved in abscisic acid biosynthesis and corresponding to the ABA locus of Arabidopsis thaliana**. *EMBO J* 1996, **15**(10):2331-2342.
60. Karssen CM, Brinkhorst-van der Swan DLC, Breekland AE, Koornneef M: **Induction of dormancy during seed development by endogenous abscisic acid: studies on abscisic acid deficient genotypes of Arabidopsis thaliana (L.) Heynh**. *Planta* 1983, **157**(2):158-165.
61. Liang X, Abel S, Keller JA, Shen NF, Theologis A: **The 1-aminocyclopropane-1-carboxylate synthase gene family of Arabidopsis thaliana**. *Proceedings of the National Academy of Sciences* 1992, **89**(22):11046-11050.
62. Jones L, Ennos AR, Turner SR: **Cloning and characterization of irregular xylem4 (irx4): a severely lignin-deficient mutant of Arabidopsis**. *The Plant Journal* 2001, **26**(2):205-216.
63. Kim S-J, Kim K-W, Cho M-H, Franceschi VR, Davin LB, Lewis NG: **Expression of cinnamyl alcohol dehydrogenases and their putative homologues during Arabidopsis thaliana growth and development: Lessons for database annotations?** *Phytochemistry* 2007, **68**(14):1957-1974.

64. Schrick K, Mayer U, Horrichs A, Kuhnt C, Bellini C, Dangl J, Schmidt J, Jürgens G: **FACKEL is a sterol C-14 reductase required for organized cell division and expansion in Arabidopsis embryogenesis**. *Genes & Development* 2000, **14**(12):1471-1484.
65. Pullen M, Clark N, Zarinkamar F, Topping J, Lindsey K: **Analysis of Vascular Development in the <italic>hydra</ italic> Sterol Biosynthetic Mutants of Arabidopsis**. *PLoS One* 2010, **5**(8):e12227.
66. Laloi M, Perret A-M, Chatre L, Melser S, Cantrel C, Vaultier M-N, Zachowski A, Bathany K, Schmitter J-M, Vallet M *et al*: **Insights into the Role of Specific Lipids in the Formation and Delivery of Lipid Microdomains to the Plasma Membrane of Plant Cells**. *Plant Physiology* 2007, **143**(1):461-472.
67. Fischer U, Men S, Grebe M: **Lipid function in plant cell polarity**. *Current Opinion in Plant Biology* 2004, **7**(6):670-676.
68. Nakashima K, Fujita Y, Kanamori N, Katagiri T, Umezawa T, Kidokoro S, Maruyama K, Yoshida T, Ishiyama K, Kobayashi M *et al*: **Three Arabidopsis SnRK2 protein kinases, SRK2D/ SnRK2.2, SRK2E/ SnRK2.6/ OST1 and SRK2I/ SnRK2.3, involved in ABA signaling are essential for the control of seed development and dormancy**. *Plant Cell Physiol* 2009, **50**(7):1345-1363.
69. Boudsocq M, Droillard M-J, Barbier-Brygoo H, Laurière C: **Different phosphorylation mechanisms are involved in the activation of sucrose non-fermenting 1 related protein kinases 2 by osmotic stresses and abscisic acid**. *Plant Molecular Biology* 2007, **63**(4):491-503.
70. Fujii H, Verslues PE, Zhu JK: **Identification of two protein kinases required for abscisic acid regulation of seed germination, root growth, and gene expression in Arabidopsis**. *Plant Cell* 2007, **19**(2):485-494.
71. Suhita D, Raghavendra AS, Kwak JM, Vavasseur A: **Cytoplasmic Alkalization Precedes Reactive Oxygen Species Production during Methyl Jasmonate- and Abscisic Acid-Induced Stomatal Closure**. *Plant Physiology* 2004, **134**(4):1536-1545.
72. Cho Y-H, Hong J-W, Kim E-C, Yoo S-D: **Regulatory Functions of SnRK1 in Stress-Responsive Gene Expression and in Plant Growth and Development**. *Plant Physiology* 2012, **158**(4):1955-1964.
73. Coello P, Hey SJ, Halford NG: **The sucrose non-fermenting-1-related (SnRK) family of protein kinases: potential for manipulation to improve stress tolerance and increase yield**. *Journal of Experimental Botany* 2011, **62**(3):883-893.
74. Baena-Gonzalez E, Rolland F, Thevelein JM, Sheen J: **A central integrator of transcription networks in plant stress and energy signalling**. *Nature* 2007, **448**(7156):938-942.
75. Baena-González E, Sheen J: **Convergent energy and stress signaling**. *Trends in plant science* 2008, **13**(9):474-482.
76. Wang H, Lockwood SK, Hoeltzel MF, Schiefelbein JW: **The ROOT HAIR DEFECTIVE3 gene encodes an evolutionarily conserved protein with GTP-binding motifs and is required for regulated cell enlargement in Arabidopsis**. *Genes & Development* 1997, **11**(6):799-811.
77. Hu Y, Zhong R, Morrison Iii WH, Ye Z-H: **The Arabidopsis RHD3 gene is required for cell wall biosynthesis and actin organization**. *Planta* 2003, **217**(6):912-921.
78. Pata MO, Hannun YA, Ng CKY: **Plant sphingolipids: decoding the enigma of the Sphinx**. *New Phytologist* 2010, **185**(3):611-630.
79. Steponkus P, Uemura M, Webb M: **A contrast of the cryostability of the plasma membrane of winter rye and spring oat-two species that widely differ in their freezing tolerance and plasma membrane lipid composition**. *Advances in low-temperature biology* 1993, **2**:211-312.
80. Noh B, Murphy AS, Spalding EP: **Multidrug resistance-like genes of Arabidopsis required for auxin transport and auxin-mediated development**. *Plant Cell* 2001, **13**(11):2441-2454.
81. Wu G, Lewis DR, Spalding EP: **Mutations in Arabidopsis Multidrug Resistance-Like ABC Transporters Separate the Roles of Acropetal and Basipetal Auxin Transport in Lateral Root Development**. *The Plant Cell Online* 2007, **19**(6):1826-1837.
82. Blakeslee JJ, Bandyopadhyay A, Lee OR, Mravec J, Titapiwatanakun B, Sauer M, Makam SN, Cheng Y, Bouchard R, Adamec J *et al*: **Interactions among PIN-FORMED and P-Glycoprotein Auxin Transporters in Arabidopsis**. *The Plant Cell Online* 2007, **19**(1):131-147.
83. Zhao H, Liu L, Mo H, Qian L, Cao Y, Cui S, Li X, Ma L: **The ATP-Binding Cassette Transporter <italic>ABCB19</ italic> Regulates Postembryonic Organ Separation in <italic>Arabidopsis</ italic>**. *PLoS One* 2013, **8**(4):e60809.

84. Barbez E, Kubes M, Rolcik J, Beziat C, Pencik A, Wang B, Rosquete MR, Zhu J, Dobrev PI, Lee Y *et al*: **A novel putative auxin carrier family regulates intracellular auxin homeostasis in plants**. *Nature* 2012, **485**(7396):119-122.
85. Ruiz Rosquete M, Barbez E, Kleine-Vehn J: **Cellular Auxin Homeostasis: Gatekeeping Is Housekeeping**. *Molecular Plant* 2012, **5**(4):772-786.
86. Baxter IR, Young JC, Armstrong G, Foster N, Bogenschutz N, Cordova T, Peer WA, Hazen SP, Murphy AS, Harper JF: **A plasma membrane H+-ATPase is required for the formation of proanthocyanidins in the seed coat endothelium of Arabidopsis thaliana**. *Proceedings of the National Academy of Sciences of the United States of America* 2005, **102**(7):2649-2654.
87. Marinova K, Pourcel L, Weder B, Schwarz M, Barron D, Routaboul J-M, Debeaujon I, Klein M: **The Arabidopsis MATE Transporter TT12 Acts as a Vacuolar Flavonoid/ H+-Antiporter Active in Proanthocyanidin-Accumulating Cells of the Seed Coat**. *The Plant Cell Online* 2007, **19**(6):2023-2038.
88. Ohashi-Ito K, Matsukawa M, Fukuda H: **An Atypical bHLH Transcription Factor Regulates Early Xylem Development Downstream of Auxin**. *Plant and Cell Physiology* 2013, **54**(3):398-405.
89. Gong Q, Li P, Ma S, Indu Rupassara S, Bohnert HJ: **Salinity stress adaptation competence in the extremophile Thellungiella halophila in comparison with its relative Arabidopsis thaliana**. *The Plant Journal* 2005, **44**(5):826-839.
90. Huang D, Wu W, Abrams SR, Cutler AJ: **The relationship of drought-related gene expression in Arabidopsis thaliana to hormonal and environmental factors**. *Journal of Experimental Botany* 2008, **59**(11):2991-3007.
91. Choi HI, Park HJ, Park JH, Kim S, Im MY, Seo HH, Kim YW, Hwang I, Kim SY: **Arabidopsis calcium-dependent protein kinase AtCPK32 interacts with ABF4, a transcriptional regulator of abscisic acid-responsive gene expression, and modulates its activity**. *Plant Physiol* 2005, **139**(4):1750-1761.
92. Weinl S, Kudla J: **The CBL–CIPK Ca2+-decoding signaling network: function and perspectives**. *New Phytologist* 2009, **184**(3):517-528.
93. Alonso JM, Hirayama T, Roman G, Nourizadeh S, Ecker JR: **EIN2, a Bifunctional Transducer of Ethylene and Stress Responses in Arabidopsis**. *Science* 1999, **284**(5423):2148-2152.
94. Beaudoin N, Serizet C, Gosti F, Giraudat J: **Interactions between Abscisic Acid and Ethylene Signaling Cascades**. *The Plant Cell Online* 2000, **12**(7):1103-1115.
95. Ghassemian M, Nambara E, Cutler S, Kawaide H, Kamiya Y, McCourt P: **Regulation of Abscisic Acid Signaling by the Ethylene Response Pathway in Arabidopsis**. *The Plant Cell Online* 2000, **12**(7):1117-1126.
96. Shibuya K, Barry KG, Ciardi JA, Loucas HM, Underwood BA, Nourizadeh S, Ecker JR, Klee HJ, Clark DG: **The Central Role of PhEIN2 in Ethylene Responses throughout Plant Development in Petunia**. *Plant Physiology* 2004, **136**(2):2900-2912.
97. Wang Y, Liu C, Li K, Sun F, Hu H, Li X, Zhao Y, Han C, Zhang W, Duan Y *et al*: **Arabidopsis EIN2 modulates stress response through abscisic acid response pathway**. *Plant Molecular Biology* 2007, **64**(6):633-644.
98. Begheldo M, Manganaris GA, Bonghi C, Tonutti P: **Different postharvest conditions modulate ripening and ethylene biosynthetic and signal transduction pathways in Stony Hard peaches**. *Postharvest Biol Technol* 2008, **48**(1):8-8.
99. Shi Y, Tian S, Hou L, Huang X, Zhang X, Guo H, Yang S: **Ethylene signaling negatively regulates freezing tolerance by repressing expression of CBF and type-A ARR genes in Arabidopsis**. *Plant Cell* 2012, **24**(6):2578-2595.
100. Vandenbussche F, Vaseva I, Vissenberg K, Van Der Straeten D: **Ethylene in vegetative development: a tale with a riddle**. *New Phytologist* 2012, **194**(4):895-909.
101. Thain SC, Vandenbussche F, Laarhoven LJJ, Dowson-Day MJ, Wang Z-Y, Tobin EM, Harren FJM, Millar AJ, Van Der Straeten D: **Circadian Rhythms of Ethylene Emission in Arabidopsis**. *Plant Physiology* 2004, **136**(3):3751-3761.
102. Etchells JP, Provost CM, Turner SR: **Plant Vascular Cell Division Is Maintained by an Interaction between PXY and Ethylene Signalling**. *PLoS Genet* 2012, **8**(11):e1002997.
103. Woeste KE, Ye C, Kieber JJ: **Two Arabidopsis Mutants That Overproduce Ethylene Are Affected in the Posttranscriptional Regulation of 1-Aminocyclopropane-1-Carboxylic Acid Synthase**. *Plant Physiology* 1999, **119**(2):521-530.

104. Wang KLC, Yoshida H, Lurin C, Ecker JR: **Regulation of ethylene gas biosynthesis by the Arabidopsis ETO1 protein**. *Nature* 2004, **428**(6986):945-950.
105. Beck M, Komis G, Müller J, Menzel D, Šamaj J: **Arabidopsis Homologs of Nucleus- and Phragmoplast-Localized Kinase 2 and 3 and Mitogen-Activated Protein Kinase 4 Are Essential for Microtubule Organization**. *The Plant Cell Online* 2010, **22**(3):755-771.
106. Trotta A, Wrzaczek M, Scharte J, Tikkanen M, Konert G, Rahikainen M, Holmström M, Hiltunen H-M, Rips S, Sipari N *et al*: **Regulatory Subunit B′ γ of Protein Phosphatase 2A Prevents Unnecessary Defense Reactions under Low Light in Arabidopsis**. *Plant Physiology* 2011, **156**(3):1464-1480.
107. Bouché N, Fait A, Zik M, Fromm H: **The root-specific glutamate decarboxylase (GAD1) is essential for sustaining GABA levels in Arabidopsis**. *Plant Molecular Biology* 2004, **55**(3):315-325.
108. Kirch H-H, Nair A, Bartels D: **Novel ABA- and dehydration-inducible aldehyde dehydrogenase genes isolated from the resurrection plant Craterostigma plantagineum and Arabidopsis thaliana**. *The Plant Journal* 2001, **28**(5):555-567.
109. Yip W-K, Yang SF: **Cyanide Metabolism in Relation to Ethylene Production in Plant Tissues**. *Plant Physiology* 1988, **88**(2):473-476.
110. Pommerrenig B, Feussner K, Zierer W, Rabinovych V, Klebl F, Feussner I, Sauer N: **Phloem-Specific Expression of Yang Cycle Genes and Identification of Novel Yang Cycle Enzymes in Plantago and Arabidopsis**. *The Plant Cell Online* 2011, **23**(5):1904-1919.
111. Van de Poel B, Bulens I, Markoula A, Hertog MLATM, Dreesen R, Wirtz M, Vandoninck S, Oppermann Y, Keulemans J, Hell R *et al*: **Targeted Systems Biology Profiling of Tomato Fruit Reveals Coordination of the Yang Cycle and a Distinct Regulation of Ethylene Biosynthesis during Postclimacteric Ripening**. *Plant Physiology* 2012, **160**(3):1498-1514.
112. Inaba K, Fujiwara T, Hayashi H, Chino M, Komeda Y, Naito S: **Isolation of an Arabidopsis thaliana Mutant, mto1, That Overaccumulates Soluble Methionine (Temporal and Spatial Patterns of Soluble Methionine Accumulation)**. *Plant Physiology* 1994, **104**(3):881-887.
113. Kitamura S, Matsuda F, Tohge T, Yonekura-Sakakibara K, Yamazaki M, Saito K, Narumi I: **Metabolic profiling and cytological analysis of proanthocyanidins in immature seeds of Arabidopsis thaliana flavonoid accumulation mutants**. *The Plant Journal* 2010, **62**(4):549-559.
114. Kitamura S, Shikazono N, Tanaka A: **TRANSPARENT TESTA 19 is involved in the accumulation of both anthocyanins and proanthocyanidins in Arabidopsis**. *The Plant Journal* 2004, **37**(1):104-114.
115. Danyluk J, Carpentier E, Sarhan F: **Identification and characterization of a low temperature regulated gene encoding an actin-binding protein from wheat**. *FEBS Letters* 1996, **389**(3):324-327.
116. Ouellet F, Carpentier É, Cope MJTV, Monroy AF, Sarhan F: **Regulation of a Wheat Actin-Depolymerizing Factor during Cold Acclimation**. *Plant Physiology* 2001, **125**(1):360-368.
117. Mao T, Jin L, Li H, Liu B, Yuan M: **Two Microtubule-Associated Proteins of the Arabidopsis MAP65 Family Function Differently on Microtubules**. *Plant Physiology* 2005, **138**(2):654-662.
118. Zhang Q, Lin F, Mao T, Nie J, Yan M, Yuan M, Zhang W: **Phosphatidic Acid Regulates Microtubule Organization by Interacting with MAP65-1 in Response to Salt Stress in Arabidopsis**. *The Plant Cell Online* 2012, **24**(11):4555-4576.
119. Steinborn K, Maulbetsch C, Priester B, Trautmann S, Pacher T, Geiges B, Küttner F, Lepiniec L, Stierhof Y-D, Schwarz H *et al*: **The Arabidopsis PILZ group genes encode tubulin-folding cofactor orthologs required for cell division but not cell growth**. *Genes & Development* 2002, **16**(8):959-971.
120. Ohta M, Ohme-Takagi M, Shinshi H: **Three ethylene-responsive transcription factors in tobacco with distinct transactivation functions**. *The Plant Journal* 2000, **22**(1):29-38.
121. Belin C, Megies C, Hauserova E, Lopez-Molina L: **Abscisic acid represses growth of the Arabidopsis embryonic axis after germination by enhancing auxin signaling**. *Plant Cell* 2009, **21**(8):2253-2268.
122. Borevitz JO, Xia Y, Blount J, Dixon RA, Lamb C: **Activation Tagging Identifies a Conserved MYB Regulator of Phenylpropanoid Biosynthesis**. *The Plant Cell Online* 2000, **12**(12):2383-2393.

123. Cominelli E, Gusmaroli G, Allegra D, Galbiati M, Wade HK, Jenkins GI, Tonelli C: **Expression analysis of anthocyanin regulatory genes in response to different light qualities in Arabidopsis thaliana**. *Journal of Plant Physiology* 2008, **165**(8):886-894.
124. Moes D, Gatti S, Hoffmann C, Dieterle M, Moreau F, Neumann K, Schumacher M, Diederich M, Grill E, Shen W-H *et al*: **A LIM Domain Protein from Tobacco Involved in Actin-Bundling and Histone Gene Transcription**. *Molecular Plant* 2012.
125. Bemer M, Karlova R, Ballester AR, Tikunov YM, Bovy AG, Wolters-Arts M, Rossetto PdB, Angenent GC, de Maagd RA: **The Tomato FRUITFULL Homologs TDR4/ FUL1 and MBP7/ FUL2 Regulate Ethylene-Independent Aspects of Fruit Ripening**. *The Plant Cell Online* 2012, **24**(11):4437-4451.
126. Jaakola L, Poole M, Jones MO, Kämäräinen-Karppinen T, Koskimäki JJ, Hohtola A, Häggman H, Fraser PD, Manning K, King GJ *et al*: **A SQUAMOSA MADS Box Gene Involved in the Regulation of Anthocyanin Accumulation in Bilberry Fruits**. *Plant Physiology* 2010, **153**(4):1619-1629.
127. Tani E, Polidoros AN, Tsaftaris AS: **Characterization and expression analysis of FRUITFULL- and SHATTERPROOF-like genes from peach (Prunus persica) and their role in split-pit formation**. *Tree Physiology* 2007, **27**(5):649-659.
128. Ferrandiz C, Liljegren SJ, Yanofsky MF: **Negative regulation of the SHATTERPROOF genes by FRUITFULL during Arabidopsis fruit development**. *Science* 2000, **289**(5478):436-438.
129. Itkin M, Seybold H, Breitel D, Rogachev I, Meir S, Aharoni A: **TOMATO AGAMOUS-LIKE 1 is a component of the fruit ripening regulatory network**. *The Plant Journal* 2009, **60**(6):1081-1095.
130. Vrebalov J, Pan IL, Arroyo AJM, McQuinn R, Chung M, Poole M, Rose J, Seymour G, Grandillo S, Giovannoni J *et al*: **Fleshy Fruit Expansion and Ripening Are Regulated by the Tomato SHATTERPROOF Gene TAGL1**. *The Plant Cell Online* 2009, **21**(10):3041-3062.
131. Giménez E, Pineda B, Capel J, Antón MT, Atarés A, Pérez-Martín F, García-Sogo B, Angosto T, Moreno V, Lozano R: **Functional Analysis of the <italic>Arlequin</ italic> Mutant Corroborates the Essential Role of the <italic>ARLEQUIN/ TAGL1</ italic> Gene during Reproductive Development of Tomato**. *PLoS One* 2010, **5**(12):e14427.
132. Berkowitz O, Jost R, Pollmann S, Masle J: **Characterization of TCTP, the Translationally Controlled Tumor Protein, from Arabidopsis thaliana**. *The Plant Cell Online* 2008, **20**(12):3430-3447.
133. Leiber R-M, John F, Verhertbruggen Y, Diet A, Knox JP, Ringli C: **The TOR Pathway Modulates the Structure of Cell Walls in Arabidopsis**. *The Plant Cell Online* 2010, **22**(6):1898-1908.
134. Devic M, Guilleminot J, Debeaujon I, Bechtold N, Bensaude E, Koornneef M, Pelletier G, Delseny M: **The BANYULS gene encodes a DFR-like protein and is a marker of early seed coat development**. *The Plant Journal* 1999, **19**(4):387-398.
135. Yamagami T, Tsuchisaka A, Yamada K, Haddon WF, Harden LA, Theologis A: **Biochemical Diversity among the 1-Amino-cyclopropane-1-Carboxylate Synthase Isozymes Encoded by the Arabidopsis Gene Family**. *Journal of Biological Chemistry* 2003, **278**(49):49102-49112.
136. Qian D, Jiang L, Lu L, Wei C, Li Y: **Biochemical and Structural Properties of Cyanases from <italic>Arabidopsis thaliana</ italic> and <italic>Oryza sativa</ italic>**. *PLoS One* 2011, **6**(3):e18300.
137. Piotrowski M, Schönfelder S, Weiler EW: **The Arabidopsis thaliana Isogene NIT4 and Its Orthologs in Tobacco Encode β-Cyano-l-alanine Hydratase/ Nitrilase**. *Journal of Biological Chemistry* 2001, **276**(4):2616-2621.
138. Shirley BW, Hanley S, Goodman HM: **Effects of ionizing radiation on a plant genome: analysis of two Arabidopsis transparent testa mutations**. *The Plant Cell Online* 1992, **4**(3):333-347.
139. Li J, Ou-Lee TM, Raba R, Amundson RG, Last RL: **Arabidopsis Flavonoid Mutants Are Hypersensitive to UV-B Irradiation**. *The Plant Cell Online* 1993, **5**(2):171-179.
140. Brown DE, Rashotte AM, Murphy AS, Normanly J, Tague BW, Peer WA, Taiz L, Muday GK: **Flavonoids act as negative regulators of auxin transport in vivo in arabidopsis**. *Plant Physiol* 2001, **126**(2):524-535.

141. Moreau M, Azzopardi M, Clément G, Dobrenel T, Marchive C, Renne C, Martin-Magniette M-L, Taconnat L, Renou J-P, Robaglia C *et al*: **Mutations in the Arabidopsis Homolog of LST8/ GβL, a Partner of the Target of Rapamycin Kinase, Impair Plant Growth, Flowering, and Metabolic Adaptation to Long Days**. *The Plant Cell Online* 2012, **24**(2):463-481.
142. Moshkov IE, Mur LAJ, Novikova GV, Smith AR, Hall MA: **Ethylene Regulates Monomeric GTP-Binding Protein Gene Expression and Activity in Arabidopsis**. *Plant Physiology* 2003, **131**(4):1705-1717.
143. Wang C, Yan X, Chen Q, Jiang N, Fu W, Ma B, Liu J, Li C, Bednarek SY, Pan J: **Clathrin Light Chains Regulate Clathrin-Mediated Trafficking, Auxin Signaling, and Development in Arabidopsis**. *The Plant Cell Online* 2013.
144. Van Damme D, Coutuer S, De Rycke R, Bouget F-Y, Inzé D, Geelen D: **Somatic Cytokinesis and Pollen Maturation in Arabidopsis Depend on TPLATE, Which Has Domains Similar to Coat Proteins**. *The Plant Cell Online* 2006, **18**(12):3502-3518.
145. Gadeyne A, Sánchez-Rodríguez C, Vanneste S, Di Rubbo S, Zauber H, Vanneste K, Van Leene J, De Winne N, Eeckhout D, Persiau G *et al*: **The TPLATE Adaptor Complex Drives Clathrin-Mediated Endocytosis in Plants**. *Cell*, **156**(4):691-704.
146. Spitzer C, Reyes FC, Buono R, Sliwinski MK, Haas TJ, Otegui MS: **The ESCRT-Related CHMP1A and B Proteins Mediate Multivesicular Body Sorting of Auxin Carriers in Arabidopsis and Are Required for Plant Development**. *The Plant Cell Online* 2009, **21**(3):749-766.
147. Tanaka H, Nodzyński T, Kitakura S, Feraru MI, Sasabe M, Ishikawa T, Kleine-Vehn J, Kakimoto T, Friml J: **BEX1/ ARF1A1C is Required for BFA-Sensitive Recycling of PIN Auxin Transporters and Auxin-Mediated Development in Arabidopsis**. *Plant and Cell Physiology* 2014, **55**(4):737-749.
148. Niemes S, Labs M, Scheuring D, Krueger F, Langhans M, Jesenofsky B, Robinson DG, Pimpl P: **Sorting of plant vacuolar proteins is initiated in the ER**. *The Plant Journal* 2010, **62**(4):601-614.
149. Kleine-Vehn J, Leitner J, Zwiewka M, Sauer M, Abas L, Luschnig C, Friml J: **Differential degradation of PIN2 auxin efflux carrier by retromer-dependent vacuolar targeting**. *Proceedings of the National Academy of Sciences* 2008, **105**(46):17812-17817.
150. Zelazny E, Santambrogio M, Pourcher M, Chambrier P, Berne-Dedieu A, Fobis-Loisy I, Miège C, Jaillais Y, Gaude T: **Mechanisms Governing the Endosomal Membrane Recruitment of the Core Retromer in Arabidopsis**. *Journal of Biological Chemistry* 2013, **288**(13):8815-8825.
151. Stepanova AN, Hoyt JM, Hamilton AA, Alonso JM: **A Link between ethylene and auxin uncovered by the characterization of two root-specific ethylene-insensitive mutants in Arabidopsis**. *Plant Cell* 2005, **17**(8):2230-2242.
152. Tian Q, Reed JW: **Control of auxin-regulated root development by the Arabidopsis thaliana SHY2/ IAA3 gene**. *Development* 1999, **126**(4):711-721.
153. Romera-Branchat M, Ripoll JJ, Yanofsky MF, Pelaz S: **The WOX13 homeobox gene promotes replum formation in the Arabidopsis thaliana fruit**. *The Plant Journal* 2013, **73**(1):37-49.
154. Vanneste S, De Rybel B, Beemster GTS, Ljung K, De Smet I, Van Isterdael G, Naudts M, Iida R, Gruissem W, Tasaka M *et al*: **Cell Cycle Progression in the Pericycle Is Not Sufficient for SOLITARY ROOT/ IAA14-Mediated Lateral Root Initiation in Arabidopsis thaliana**. *The Plant Cell Online* 2005, **17**(11):3035-3050.
155. Jin H, Cominelli E, Bailey P, Parr A, Mehrtens F, Jones J, Tonelli C, Weisshaar B, Martin C: **Transcriptional repression by AtMYB4 controls production of UV‐protecting sunscreens in Arabidopsis**. *The EMBO Journal* 2000, **19**(22):6150-6161.
156. Hua J, Sakai H, Nourizadeh S, Chen QG, Bleecker AB, Ecker JR, Meyerowitz EM: **EIN4 and ERS2 Are Members of the Putative Ethylene Receptor Gene Family in Arabidopsis**. *The Plant Cell Online* 1998, **10**(8):1321-1332.
157. Tieman DM, Taylor MG, Ciardi JA, Klee HJ: **The tomato ethylene receptors NR and LeETR4 are negative regulators of ethylene response and exhibit functional compensation within a multigene family**. *Proceedings of the National Academy of Sciences* 2000, **97**(10):5663-5668.
158. Woeste KE, Kieber JJ: **A Strong Loss-of-Function Mutation in RAN1 Results in Constitutive Activation of the Ethylene Response Pathway as Well as a Rosette-Lethal Phenotype**. *The Plant Cell Online* 2000, **12**(3):443-455.
